# Differences among domestic chicken breeds in tonic immobility responses as a measure of fearfulness

**DOI:** 10.7717/peerj.14703

**Published:** 2023-04-04

**Authors:** Inga Tiemann, Senta Becker, Jocelyn Fournier, Daalkhaijav Damiran, Wolfgang Büscher, Sonja Hillemacher

**Affiliations:** 1 Institute of Agricultural Engineering, University of Bonn, Bonn, Germany; 2 Department of Animal & Poultry Science, University of Saskatchewan, Saskatoon, Canada

**Keywords:** Fear, Chicken, Animal genetic resources, Poultry, Tonic immobility, Animal welfare

## Abstract

**Background:**

One priority for animal welfare is for animals to experience less fear, especially during human contact. For domestic animals, breeds that are less fearful may provide genetic resources to develop strains with improved welfare due to lower susceptibility to fear. Genetic predispositions inherited in these breeds might reflect the large diversity of chicken breeds. The goal of the present study was to systematically test a diverse group of chicken breeds to search for breeds that experience less fear.

**Methods:**

Nineteen chicken breeds from commercial hybrid lines, native layer-type, meat-type and dual-purpose breeds, ornamental breeds as well as bantam breeds were tested in a standardized tonic immobility (TI) test. Chickens were manually restrained on their back, and the time to first head movement and first leg movement, the duration of TI, as well as the number of attempts needed to induce TI were measured.

**Results:**

The TI response differed among chicken breeds (*p* ≤ 0.001) for naïve, mature hens. The median number of attempts required to induce TI ranged from 1 to 2 and did not differ significantly among breeds. Median durations were much more variable, with Lohmann Brown showing shortest durations (6 s, 12 s, 58 s for time to first head movement, first leg movement and total duration of TI, respectively). In contrast, medians reached the maximum of 600 s for all three measures in German Creepers. Repeated tests on the same individuals did not affect attempts needed to induce TI nor TI durations. Breeds clustered into two main groups, with layer-type native breeds and ornamental breeds having longer TI durations, and bantam, dual-purpose and meat-type native breeds having shorter TI durations.

**Conclusions:**

Our findings provide evidence for substantial variation of fearfulness among breeds. This variation could be linked to the intended use during the breed’s specific history. Knowledge and quantitative measurement of these behavioural responses provide the opportunity to improve welfare through selection and future breeding.

## Introduction

Minimizing fear is important to farm animal welfare and understanding genetic variation in the susceptibility to fear can contribute to this process. Farm animals live in an environment that is highly influenced by humans. During their lives, animals should be as free as possible from chronic and excessive fear, which has a negative impact on their well-being (Fraser, 2008; Mellor, 2016). In addition to the environmental influences that affect the behavioural response, the susceptibility to experiencing fear also has a fundamental genetic component (Jones, Mills & Faure, 1991; Boissy et al., 2005). Genetic selection to reduce fearfulness has been shown effective for livestock (Boissy et al., 2005) as well as in poultry (Agnvall et al., 2012). Knowledge of the genetic variation in susceptibility to fear may be useful if breeding for decreased fearfulness (Forkman et al., 2007; Jensen, 2014; Lord et al., 2020).

The domestic chicken has become a very diverse population since the bird’s domestication (Wood-Gush, 1959; Crawford, 1990). Breeds and overarching categories of chicken were already reported in Darwin’s (1868) “Variation of Animals and Plants under Domestication”. While egg and meat production rely on a few hybrid lines, globally there are 500 to 1,600 local and purebred chicken breeds (Qanbari et al., 2019; Food and Agriculture Organization of the United Nations, 2022), which have only a marginal impact on food production and are recognized as being genetic resources (Sørensen, 2010; Tiemann, Hillemacher & Wittmann, 2020). Some of these native breeds could have advantageous traits for climate adaptation (Mpenda et al., 2018), specific performance (Dalal, Ratwan & Yadav, 2022), and nutritional quality (Dalle Zotte et al., 2019). Typically, breeds are assigned to categories that cover bantams, based on their small body size, hybrids used for the commercial production for eggs and meat, native breeds that are differentiated into layer, meat, and dual-purpose types that originated in and are adapted to specific regions, and ornamental breeds (Crawford, 1990; Malomane et al., 2021). These categories are found in the historical and popular science literature, based on the breeds’ genetic relationship, intended use and overarching breeding objective (Dürigen, 1932; Barber, 2012; Bortoluzzi et al., 2018). These breeding objectives may include selection for tameness and the tendency to approach humans (Agnvall et al., 2015) as well as the selection of other traits, such as foraging behaviour and exploration but not proximity to humans (Agnvall et al., 2012). Breeds that are usually exposed to intensive human contact, such as bantam or hybrid breeds, are expected to be less fearful than breeds that are traditionally kept in free-range systems with less human contact, such as layer-type native breeds. In a study of Japanese breeds, dual-purpose breeds showed particularly low fear in a handling test (Moroi et al., 2019). Most chickens globally are part of commercial breeding programs that are focused on egg or meat performance and health with only minor but increasing attention toward behavioural traits (*e.g.*, Ellen et al., 2019; Brito et al., 2020; Ferreira, Guesdon & Calandreau, 2021).

A number of tests to quantify the susceptibility to fear have been proposed. Among these are general activity in open field (Tiemann et al., 2022) and proximity to a novel object (Forkman et al., 2007), struggling during restraining or handling (Jones & Faure, 1981b; Moroi et al., 2019), response to a predator (Miller, Garner & Mench, 2006), and tonic immobility (TI) tests.

The TI test is particularly useful to measure fear. The TI reaction is assumed to reflect an antipredator strategy in which an animal falls into a sustained muscle tone, and the immobility is intended to confuse and avert the attacker (Gallup, 1977). It has been reported as having high repeatability and validity, when following a standardized test procedure (Jones, 1986; Forkman et al., 2007). The repeatability and consistency of TI provides a route to search for genetic patterns in test outcomes (Jones, 1988; Ghareeb et al., 2008). The TI test for chickens was initially described by Jones & Faure (1981b) to investigate the reaction of chickens toward regular handling in terms of fear reduction. The duration of TI is correlated with the level of general fearfulness, and the primary variables measured are the time to first head and leg movement, time until righting and the number of attempts needed to induce TI. Animals that are more susceptible to fear remain in the supine position typical of TI longer and straighten up later (with a common consensus to limit TI to 600 s) than animals that are less fearful. Fearful animals also need fewer attempts to induce TI (Jones & Faure, 1981a). The number of attempts to induce TI and duration of TI have been found to be slightly negatively correlated with each other, so it can be assumed that attempts and duration are derived from different underlying mechanisms (Edelaar et al., 2012). The TI response has been used as an indicator of fearfulness in studies ranging from the impact of the environment (Gallup, Nash & Ellison, 1971; Hocking, Jones & Picard, 2005) to the influence of genetics (Jones & Faure, 1981a; Fogelholm et al., 2019). This genotype expressing an individual phenotype might result in individual free-range use (Stadig et al., 2017) or individual social motivation (Caliva et al., 2019). Although congruency among types of tests *e.g.*, open field and TI, is not high because each test causes specific behavioural responses (Campbell, Dickson & Lee, 2019), the general validity of each test is undoubted (Forkman et al., 2007).

Many environmental variables affect measures of fear in chickens. The TI response, given as number of attempts to induce TI and TI duration, is influenced by different environmental variables (Jones, 1996). These include the hatching and rearing conditions (*e.g.*, dark brooders reduce fear, Riber & Guzman, 2016; Giersberg et al., 2020), husbandry system, especially enrichment (*e.g.*, elevated platforms reduce fear, Baxter, Bailie & O’Connell, 2019; Adler et al., 2020), and outdoor access (Mahboub, Müller & von Borell, 2004; Shimmura et al., 2010; Hartcher et al., 2016). In these studies, chickens with high free-range use showed lower TI duration, although others have not found such a correlation (Campbell et al., 2016; Campbell, Dickson & Lee, 2019; Bari et al., 2021). Associations with TI have also been found for social environment (Bilčık, Keeling & Newberry, 1998), nutrition (Hocking, Jones & Picard, 2005), light management (Sobotik, Nelson & Archer, 2020), bedding (El-Lethey et al., 2000), noise (Campo, Gil & Davila, 2005; Flores et al., 2019) and age of the animals (Rovee & Kleinman, 1974). These environmental variables need to be controlled to identify genetic effects.

Genetic variables have been shown to influence fear in chickens. White laying hens show longer TI responses than brown laying hens (Albentosa, Kjær & Nicol, 2003; Mahboub, Müller & von Borell, 2004; Fraisse & Cockrem, 2006), with rare exceptions (Hocking et al., 2001; Peixoto et al., 2020). Campo et al. (2007) found significant differences between Spanish and hybrid breeds in their TI duration, although their study was focused on lighting management and induced stress response, and the authors did not discuss breed differences. These studies, the establishment of quail lines (*Coturnix coturnix japonica*) selected for short and long TI duration, and genomic studies show that TI responses, and consequently fearfulness, have a genetic basis with a high heritability of 50–90% (Jones, Mills & Faure, 1991; Fogelholm et al., 2019; Ishikawa et al., 2020). Although the relationship between genetics and the fear response has been demonstrated repeatedly for the domestic chicken (Gallup, 1974; Fogelholm et al., 2019), only a small number of studies have focused on the breed-specific behavioural phenotypes, especially those exhibited by native chickens (Desta, 2019; Krause et al., 2019; Drozdová et al., 2021). Among native chickens, different TI responses are expected for breeds with different breeding backgrounds. Linking these breeding backgrounds to fear responses may offer new insights into the utilization of animal genetic resources. The diversity of breeds and their breed-specific behavioural responses contrast with the few highly specialized and inbred performance lines. However, to achieve high animal welfare standards through breeding, we need to know the quantitative variation of fear susceptibility in the first place, especially when linked to genetic predispositions.

Adaptative responses to a fear-provoking environment might be driven by individual behavioural changes such as habituation. Habituation is a general learning mechanism that results in the reduction of stress and fear as the individual gathers positive information in a repeating situation; alternatively, negative information will increase stress and fear (Canario et al., 2013; Mota-Rojas et al., 2020; Schillings, Bennett & Rose, 2021). The predisposition to habituate to fear-eliciting environmental conditions might therefore be of high interest for animal welfare and related breeding strategies (Brito et al., 2020).

The objectives of our study were to measure the quantitative variation of the TI response as an indicator of fear in different categories of domestic chicken breeds. The variables assessed were the total duration of TI response, the time to first head and first leg movement, and the number of attempts needed to induce TI, investigated among 19 chicken breeds. Following Edelaar et al. (2012), we analysed the number of attempts needed to induce TI and the duration of TI separately, assuming that these are variables influenced by different underlying mechanisms. Assuming high repeatability, we also tested whether any of the TI response traits are susceptible to habituation. Our specific goals were to examine (1) whether the number of attempts required to induce TI and the duration of TI varied among breeds, (2) whether variation among breeds in number of attempts and duration correlated with each other, (3) whether variation among breeds in measures of TI was related to the categories of chicken breeds, and (4) whether chickens showed habituation for any of the measures over three repetitions of TI induction. We hypothesized that selection has altered fearfulness in domestic chickens, resulting in breed-specific ranges of TI responses.

## Materials & Methods

### Ethics statement

Animal husbandry complied with the order on the protection of animals and the keeping of production animals (Tierschutz-Nutztierhaltungsverordnung, 2006; last revision 2017). The testing procedure was approved by the responsible authority (North Rhine-Westphalia State Agency for Nature, Environment and Consumer Protection, AZ 81-02.04.2019.A372).

### Animals

Nineteen different breeds of six categories were tested: bantam (four breeds), commercial hybrids (four lines), native layer-type (three breeds), native meat-type (one breed), native dual-purpose (three breeds), and ornamental (four breeds). Chickens were assessed using a standardized TI test, which focused on fear behaviour in two sets of chickens: Part A) mature and experimentally naïve hens (*N* = 178) and Part B) a subset of A based on 15 breeds, hens with three measurements of induced TI (Part B, *N* = 117). The breeds, additional information on the number of animals per breed tested (at least five individuals), their average weight and age are provided in Table 1. All chickens were individually identified by coloured leg bands. Chickens were housed at the Poultry Research Centre, Rhein-Kreis-Neuss, Germany. Inclusion of endangered breeds limited our ability to use animals of the same age and rearing conditions. Therefore, the study included some breeds where the birds were raised at the Centre and others which were purchased from breeders in Germany and the Netherlands. Hens raised on site were not tested before reaching sexual maturity at 21 weeks, and those obtained from external breeders were kept on site for at least 6 months before testing. Hens ranged from 21 weeks to 6 years of age at testing. After the tests, chickens remained on farm for breeding purposes or were given to private breeders who support the preservation of animal genetic resources.

**Table 1:** Breeds of chickens tested for Tonic Immobility (TI), grouped in the categories bantam, hybrids, native and ornamental breeds (in alphabetical order). The table shows sample sizes [n] for the first (A) and second plus third (B) induction of TI, mean weight and mean age ± SD at testing. LSL Classic refers to Lohmann Selected Leghorn Classic, and the abbreviation Berg. refers to Bergischer which is a prefix for Schlotterkamm as well as Long Crower.

**Category**		**Breed**	**Sample size (n)**		**Weight (g)**	**Age (weeks)**
			First induction of TI (Part A)	Second and third induction of TI (Part B)		
**Bantam breeds**		Bantam Silkie	7	7	546 ± 74	59 ± 7
		Japanese bantam	16	14	634 ± 58	62 ± 5
		Ohiki	9	9	606 ± 59	58 ± 6
		Rosecomb bantam	6	0	517 ± 23	191 ± 41
**Hybrid lines**		Cobb 500 breeder	5	5	5,513 ± 252	52 ± 0
		Lohmann Brown	10	0	1,571 ± 114	21 ± 0
		Lohmann Dual	7	0	1,547 ± 150	22 ± 1
		LSL Classic	16	6	1,624 ± 148	33 ± 4
**Native breeds**	**Layer-type**	Berg. Schlotterkamm	17	5	1,606 ± 312	31 ± 4
		East Frisian Gull	6	6	1,883 ± 102	52 ± 0
		Leghorn	6	6	2,720 ± 275	52 ± 0
	**Meat-type**	Cochin	12	11	2,738 ± 335	74 ± 8
	**Dual-purpose**	Breda	8	6	1,823 ± 259	65 ± 9
		German Empire Breed	10	0	1,631 ± 188	22 ± 1
		Marans	5	5	2,195 ± 124	62 ± 4
**Ornamental breeds**		Berg. Long Crower	9	9	2,074 ± 346	116 ± 21
		German Creeper	10	10	1,651 ± 130	52 ± 0
		Poland	13	13	1,588 ± 279	84 ± 16
		Yokohama	6	5	1,662 ± 263	173 ± 40

### Housing

All hens of the same breed were kept in a stable social group with one or two roosters in a wooden coop of 6 m^2^ with perches, nests, and bedding of wood shavings. All chickens had outdoor access to a free-range area of 200 m^2^ every day. Chickens could choose between the barn and free-range areas but spent most of the day outside, entering the barn mainly to feed, drink, or lay. Chickens were confined from dusk to dawn for safety reasons using an automatic door with light sensor (Axt Elektronic, Eisenach, Germany). The outside “stocking density” ranged from 12 m^2^ per animal for Bergische Schlotterkamm to 40 m^2^ per animal for Marans. Visual inspections, including visual health checks, and the removal of manure from the main area underneath the perches were conducted daily. A 12L:12D artificial light program ensured the same light conditions within coops throughout the year, although all chickens also had access to natural daylight in the free-range area every day.

A conventional feeding program was applied: starter and pre-layer food diets were used during growth while mature chickens were fed Deuka allmash “Zucht” pellets with a composition of crude protein 16.5%; methionine 0.4%; calcium 3.6%; phosphorus 0.5%; and MJ ME 12.4/kg with no coccidiostat (Deutsche Tierhaltung Cremer, Düsseldorf, Germany). Animals had ad libitum access to feed, water, and grit at all times. In instances of terminally ill chicks, a veterinarian was consulted. Chickens were vaccinated against Newcastle Disease at three-month intervals.

### Handling

Chickens were caught at least every second week to check their health, performance, weight, and outer appearance. As they were used to gentle handling, less than 2 min per bird was required to capture the chickens for the TI test. In general, chickens were carried following the guidelines of Herborn et al. (2015), in an upright position and close to the body, which has been shown to be less stressful for them. For TI testing, chickens were pseudo-randomly chosen from each group, ensuring that none of the chickens were caught more than once per day, and transported to the testing room using boxes of 80*40*50 cm (length*width*height) with a wire-protected cut-out to allow the birds access to light (instead of being enclosed in full darkness). Four boxes at a time were carried carefully, for not more than 80 m, on a small wagon, limiting the time each animal stayed in a box to a maximum of one hour. Two necessary procedures were weighed against each other: multiple entries and individual capture on the one hand and a short stay in the transport box on the other, with the latter considered to have a lower stress factor. The testing schedule ensured that time spent in a box was balanced across all individuals of a given breed.

### Experimental setup

Animals were placed in an arena measuring 180*180 cm. The height of the walls was 72 cm. The floor and walls were made of grey Trovidur^®^ (unplasticized PVC; Kümpel Kunststoff Verarbeitungswerk GmbH, Frankenthal, Germany). During testing, the floor was covered with a green water-resistant vinyl film. The room where the arena was housed had a daylight-emitting fluorescent tube on the ultraviolet (UV)-spectrum (Arcadia Products, Redhill, United Kingdom) and an electronic ballast unit (Relco Group Germany GmbH, Hilden; Germany) to increase flicker frequency. The light was adjusted to meet the animal’s requirements of more than 87 Hz and UV components of light (Lisney et al., 2011). A camera was hung above the arena (Eyseo EcoLine TV 8750 provided by ABUS Security Center GmbH & Co. KG, Affing, Germany), which was connected to a computer through an Advanced Dv Converter 55 (Canopus Co, Ltd., Zhejiang, China). Videos were recorded and shown in an adjacent room using “Team-Viewer” 5.1.10408 (Team Viewer GmbH, Göppingen, Germany) so that the animals were not disturbed during testing.

### Experimental procedure

Considering their diurnal behaviour, animals were only tested during the day. Chickens of all breeds were tested over the course of two years in pseudo-random order and throughout the year, depending on the availability of breeds and adult hens.

The basic procedure of the TI test was in line with Jones & Faure (1981a); Jones & Faure (1981b) and followed Hocking et al. (2001). Because breeds with different weights and morphologies were tested, no cradle was used in the TI procedure. During the TI test, a hen was carried into the arena and carefully turned onto her back on the floor with her wings close to the body. The chicken was restrained in this position for 15 s, after which the experimenter removed their hand from the animal’s sternum and left the room. The chicken remained in the arena and the human remained outside the room until the chicken showed the righting response, indicated by standing on both feet in an upright position. The TI response was considered to have been successfully induced if the animal stayed in the inverted position for at least 10 s after the experimenter removed their hand. All time measurements started with lifting the hand from the animal’s sternum which was recorded by video. If the animal got up before 10 s had passed, then the induction was considered as having failed. Failed inductions were attempted up to two times immediately afterwards. The number of attempts to induce TI was limited to three attempts per session (1 session per day). The number of sessions was not limited, resulting in a maximum of 38 attempts in 13 consecutive sessions for one hen of the breed Ohiki. After a hen got up on her feet and moved around the arena, she was taken out and weighed using a Kern HDB hand scale (Kern und Sohn GmbH, Bahlingen, Germany). If the animal did not get up after 10 min (600 s), the test was terminated, and the experimenter put the animal back on its feet. For Part A of the study, only first TI values were recorded and analysed. For a subset of animals (Part B), the TI test was repeated until three TI values had been collected in the same way as the first TI value by testing the animal a maximum of three times a day on a five days per week schedule.

The video recordings were analysed using software Viewer 3.0.1.241 (BIOBSERVE GmbH, Bonn, Germany). Exact values were used for the statistical analysis, and they were calculated by stopping the video and recording the time at first head movement after restraint, first leg movement after restraint, and righting (both feet on ground and upright body position) of the individual after restraint. Additionally, the number of unsuccessful attempts preceding the successful trial was recorded. Video analysis was carried out by the same two people who had conducted the trials. Agreement between the researchers on the measurement of the variables was established before beginning the tests.

### Data analysis

The analysis of data followed two statistical approaches:

In Part A, 178 mature, naïve hens were tested by analysing the fixed effect of breed and random effect of age (independent variables) on the number of attempts needed to induce TI (dependent variable) using a generalized linear model (GLM). The effect of the same independent variables on the three duration measures, first head movement, first leg movement and total duration of TI was analysed using three separate linear models (LM). Prior to running the models, a univariate analysis of variance (Welch-ANOVA) identified a strong association between breed and weight (F(18, 46.610) = 316.094, *P* < 0.001), so weight was excluded as independent variable from the models. Further, for each effect analysed in the models, an ANOVA for global *p*-values was calculated and followed by Holm–Bonferroni *post-hoc* tests if the ANOVA was statistically significant. A Kruskal–Wallis-test was used to compare TI total duration based on the individual data among the independent six breed categories, followed by pairwise comparisons using Holm-Bonferroni *post-hoc* tests.

In Part B, three repetitions of TI in 117 mature, naïve hens of 15 breeds were tested for the repeatability of the TI response. Here, the fixed effects repetition and breed, and the random effects age and animal’s individual identity (independent variables) on the number of attempts to induce TI (dependent variable) were assessed using a generalized linear mixed-effect model (GLMM). Time to first head movement, first leg movement and duration of TI (dependent variables) were investigated using linear mixed-effect models (LMM). Global *p*-values were calculated for each effect included in the models. When there were significant effects, pairwise comparisons using Holm-Bonferroni *post-hoc* tests were conducted. The consistency of the TI measures between individuals and within individuals over the three repetitions was tested by Kendall’s coefficient of concordance. Consistency scales ranged from 0 –1, with values above 0.7 indicating a high consistency (compare Ghareeb et al., 2008).

In both parts, dependent variables were transformed to reach normality, which was checked using a qq-plot. According to the qq-plot, the number of attempts was Poisson transformed (checked for overdispersion), time to head and leg movement were square-root transformed, and for total duration a log transformation was applied. Data are presented in the text as median (Mdn) and lower and upper quartiles (given as interquartile range IQR). In case of models with significant global *p*-values, pairwise comparisons are given in the supplemental tables (Part A: Table S1 & Part B: Table S3).

Correlations between the number of attempts and all three duration measures were analysed on the whole dataset and within breeds (and within repetition) using a two-tailed Spearman rank correlation test. We used a two-sided test with a given power of 0.8, a level of significance of 0.05 and the correlation coefficient of the corresponding correlations within breeds (Part A: Table S2 & Part B: Table S4). Therefore, we ran *post-hoc* power analyses with G*Power (G*Power Version 3.1.9.4; University Kiel, Kiel, Germany) to calculate if we met minimum required sample sizes to detect significant effects within breeds (Table S5) on the raw data which are also provided (Table S6). Data were visualized using Sigma Plot 14 (Systat Software Inc., Chicago, IL, USA). Statistical analyses were run using SPSS^®^ Statistics 27 (IBM Corporation, Armonk, NY, USA) and R 4.0.3 (R Core Team, 2019) with lme4-package for calculating the models, car package for global *p*-values and emmeans-package for pairwise comparisons as well as SAS 9.4 (Proc Mixed model procedure, SAS Institute Inc., Cary, NC, USA).

## Results

### Breed effects (Part A)

**Figure 1:**
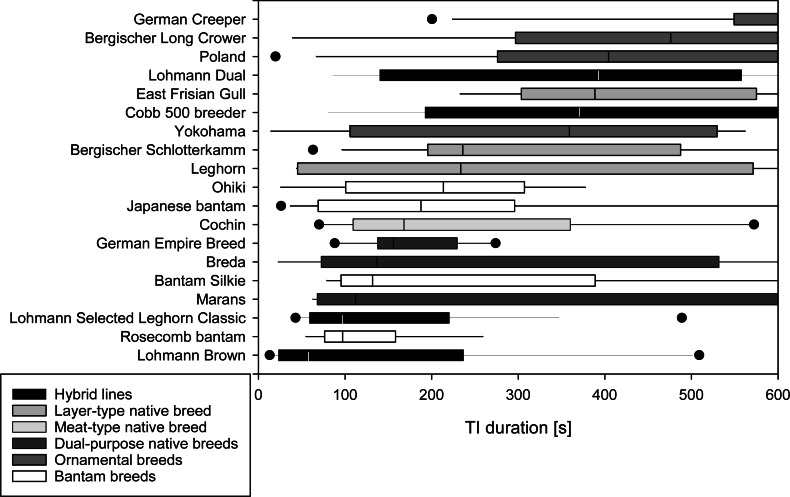
Duration of Tonic Immobility response of chicken breeds. Total duration of the tonic immobility (TI) response of 19 breeds of domestic chickens (part A; mature, experimentally naïve hens). Breeds are sorted by median duration. Medians [s] are indicated by a vertical line, 25th and 75th percentiles form the bar, 10th and 90th percentiles are indicated by whiskers, and outliers are shown as points. Grey shading reflects the categorization of the breeds based on the intended use and breeding history.

A median of 1 attempt was needed to induce TI (IQR 1-1) in the majority of the 19 breeds tested. In Rosecomb bantam, Ohiki and German Empire Breed, inductions required medians of 1.5 to 2.0 attempts (Table 2). The median time to first head movement occurred after 113 s (IQR 46–289 s) with Leghorn, Lohmann Brown, and LSL Classic showing short durations (6 to 50 s), and Berg. Long Crower, Cobb 500 breeder, and German Creeper showing long durations (369 to 600 s). The median time to first leg movement followed at 155 s (IQR 72.75–358.75 s). Among the breeds with shorter durations were Japanese Bantam, Leghorn, and Lohmann Brown (12 to 60 s), while Berg. Long Crower, Cobb 500 breeder, and German Creeper showed longer durations (378 to 522 s). The median duration of TI was 230 s (IQR 98.25-474 s), with Lohmann Brown, Lohmann Selected Leghorn Classic, and Rosecomb bantam showing short durations (58 to 98 s) and Berg. Long Crower, German Creeper and Polands showing a prolonged TI duration (404 to 600 s), with 600 s being the maximum duration of TI (Fig. 1).

**Table 2:** TI responses of 19 breeds of domestic chickens tested in Part A of the study (mature, experimentally naïve hens). Median [IQR] number of attempts required to induce Tonic Immobility (TI), latency to first head and first leg movement and the total duration of TI. For each measure, the three (and equivalent) phenotypes with the highest (H) and lowest (L) values are indicated. Note that high fear responses are indicated by low numbers of attempts and high latencies and durations. See Table 1 for sample sizes and breed abbreviations and Table 3 for statistics.

**Category**		**Breed**	**Number of attempts (n)**	**First head movement (s)**	**First leg movement (s)**	**Duration of TI (s)**
**Bantam breeds**		Bantam Silkie	1.0 [1.0-2.0]	115.0 [83.0-175.5]	131.0 [90.0-320.0]	132.0 [98.5-330.5]
		Japanese bantam	1.0 [1.0-1.3]	63.0 [41.3-131.5]	59.5 [45.8-252.3]^L^	187.5 [74.3-293.3]
		Ohiki	2.0 [1.0-2.0]^H^	78.0 [56.0-183.0]	105.0 [23.0-213.0]	214.0 [122.0-284.0]
		Rosecomb bantam	1.5 [1.0-3.5]	54.0 [53.3-62.3]	96.0 [85.0-110.8]	97.0 [86.8-118.5]^L^
**Hybrid lines**		Cobb 500 breeder	1.0 [1.0-1.0]^L^	369.0 [289.0-600.0]^H^	370.0 [299.0-600.0]^H^	372.0 [304.0-600.0]
		Lohmann Brown	1.0 [1.0-1.0]^L^	6.0 [2.5-7.0]^L^	12.0 [6.5-40.3]^L^	58.0 [27.5-155.8]^L^
		Lohmann Dual	1.0 [1.0-1.0]^L^	75.0 [59.0-127.5]	217.0 [134.5-249.0]	393.0 [187.5-476.5]
		LSL Classic	1.0 [1.0-1.0]^L^	50.0 [23.5-69.8]^L^	70.5 [45.0-130.8]	97.5 [63.0-185.5]^L^
**Native breeds**	**Layer-type**	Berg. Schlotterkamm	1.0 [1.0-1.0]^L^	178.0 [95.0-245.0]	209.0 [117.0-238.0]	236.0 [197.0-449.0]
		East Frisian Gull	1.0 [1.0-1.8]	222.0 [85.5-289.5]	193.0 [149.5-337.0]	389.0 [341.8-523.3]
		Leghorn	1.0 [1.0-1.8]	11.5 [9.5-33.8]^L^	42.5 [25.0-294.0]^L^	233.5 [53.5-519.3]
	**Meat-type**	Cochin	1.0 [1.0-1.0]^L^	84.0 [46.3-175.3]	126.0 [88.5-174.8]	168.0 [131.8-272.0]
	**Dual-purpose**	Breda	1.0 [1.0-1.0]^L^	135.0 [77.5-394.5]	135.0 [86.5-393.8]	136.5 [92.8-395.3]
		German Empire Breed	2.0 [1.0-3.0]^H^	149.0 [122.5-166.5]	155.0 [106.8-228.8]	155.5 [145.0-229.0]
		Marans	1.0 [1.0-1.0]^L^	87.0 [55.0-600.0]	95.0 [56.0-600.0]	112.0 [73.0-600.0]
**Ornamental breeds**		Berg. Long Crower	1.0 [1.0-1.0]^L^	474.0 [171.0-600.0]^H^	475.0 [172.0-600.0]^H^	476.0 [420.0-600.0]^H^
		German Creeper	1.0 [1.0-1.0]^L^	600.0 [424.5-600.0]^H^	600.0 [546.8-600.0]^H^	600.0 [589.5-600.0]^H^
		Poland	1.0 [1.0-1.0]^L^	271.0 [118.0-600.0]	298.0 [204.0-600.0]	404.0 [282.0-600.0]^H^
		Yokohama	1.0 [1.0-1.0]^L^	309.5 [139.3-489.5]	358.5 [163.5-505.5]	359.0 [164.5-506.3]

There was no significant impact of breed (*p* = 0.443) or age (*p* = 0.592) on the number of attempts needed to induce TI. There were, however, significant differences among breeds in their time for the first head movement (F(19,157) = 4.406, *p* ≤ 0.001, *R*^2^ = 0.348, R^2^ adjusted = 0.269) and first leg movement (F(19,158) = 3.731, *p* ≤ 0.001, *R*^2^ = 0.310, R^2^adjusted = 0.227) as well as for the duration of TI (F(19,158) = 3.173, *p* ≤ 0.001, *R*^2^ = 0.276, R^2^ adjusted = 0.189, Table 3, Table S1). Age did not affect the time to first head or first leg movement or the total duration of TI (all *p* ≥ 0.112, Table 3).

In 12 of the 19 breeds, the total duration of TI was significantly correlated with the time to first head movement. This correlation was even more pronounced for the time to first leg movement which was significantly correlated with the total duration of TI in 17 out of 19 breeds. The correlation of the number of attempts with total TI duration was, in contrast, only found in three out of 19 breeds (Table S2).

**Figure 2:**
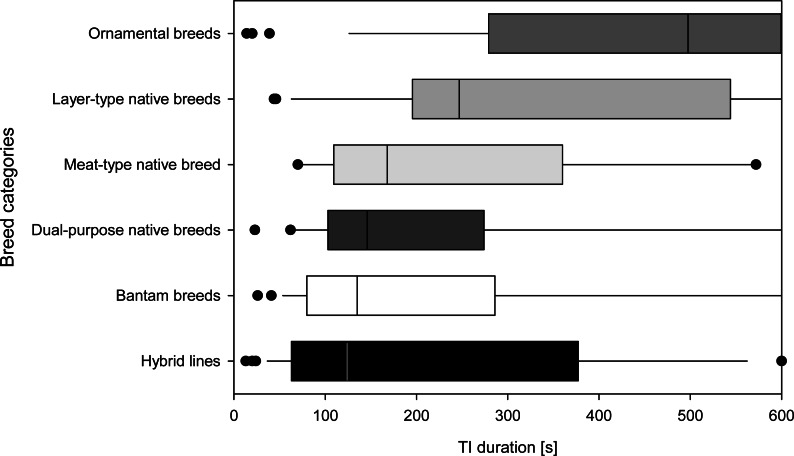
Breed categories based on TI responses. Total duration of the tonic immobility (TI) response of all individuals in each of the six breed categories (Part A; mature, experimentally naïve hens). Breed categories are sorted by median duration. Medians [s] based on all individuals in a given breed category are indicated by a vertical line, 25th and 75th percentiles form the bar, 10th and 90th percentiles are indicated by whiskers and outliers are shown as points.

When comparing the duration of TI among categories of commercial hybrid lines, native layer-type, native meat-type, native dual-purpose type, ornamental, as well as bantam breeds, the Kruskal–Wallis test showed significant differences (*N* = 178, X^2^(5) = 30.982, *p* ≤ 0.001). These differences among categories were mainly based on the ornamental breeds differing from bantam, dual-purpose native breeds and hybrid lines (all *p* ≤ 0.009, other pairwise comparisons *p* ≥ 0.124). The hybrid lines had the shortest times for TI duration, while the bantam, dual-purpose, meat- and layer-type native breeds had longer TI durations, followed by ornamental breeds which stand out statistically with the longest duration of TI (Fig. 2). The categories did not differ in the number of attempts required to induce TI (*N* = 117, F(5,111) = 0.22, *p* = 0.951).

**Table 3:** Analysis of TI responses of Subset A (naive, mature hens). The analysis of the tonic immobility (TI) response of naïve, mature hens of different breeds in Part A and for repeated inductions in Part B of the study. Reported are the type of model, the transformation to achieve normality of the dependent variables, as well as the sample size (N), median and interquartile range (IQR) of first, second and third TI and *p*-values for the number of attempts needed to induce TI, the first head movement, the first leg movement, the duration of TI. Refer to Tables S1–S4 for detailed statistics including pairwise comparisons.

		**Number of attempts**	**First head movement**	**First leg movement**	**Duration of TI**
**Part A**	Model	GLM	LM	LM	LM
	Transformation	Poisson	square-root transformed	square-root transformed	log-transformed
	N	178	177	178	178
	First TI	1 (1–1)	128.3 (53.4–528.8)	206.8 (78.2–560.8)	282.2 (103.1-581.1)
	Breed	0.443	≤ 0.001^***^	≤ 0.001^***^	≤ 0.001^***^
	Age	0.592	0.849	0.617	0.112
**Part B**	Model	GLMM	LMM	LMM	LMM
	Transformation	Poisson	log-transformed	log-transformed	square-root transformed
	N	117	107	114	117
	Second TI	1 (1–1)	105.4 (27.3–445.1)	142.5 (59.7–515.7)	197.4 (100.5-512.2)
	Third TI	1 (1–1)	190.5 (40.6–600.0)	216.2 (52.2–600.0)	295.5 (119.2-600.0)
	Breed	0.008^**^	≤ 0.001^***^	≤ 0.001^***^	≤ 0.001^***^
	Repetition	0.686	0.168	0.551	0.167

**Notes.**

* *p* ≤ 0.05.

** *p* ≤ 0.01.

*** *p* ≤ 0.001.

GLM Generalized Linear Model LM Linear Model GLMM Generalized Linear Mixed Model LMM Linear Mixed Model

### Repeated testing and breed effects (Part B)

Across the 15 breeds tested, the median number of attempts remained at 1 (IQR all 1) for all three repetitions. TI durations decreased from the first to the second repetition (1st 282 s (IQR 103-581 s); 2nd 197 s (IQR 100-512)), however, they increased again from the second to the third repetition (3rd 295 s (IQR 119-600)). There was no statistically relevant difference between these repetitions (all *p* ≥ 0.295).

Neither the duration of TI nor the attempts needed to induce TI were impacted by repeated measurements (both, *p* ≥ 0.167). In line with Part A, time to first head movement, first leg movement, and duration of TI across all repetitions were all influenced by breed (all *p* ≤ 0.001, Table 3, Table S3). The impact of breed was also found for the number of attempts needed to induce TI (*p* = 0.008)

For all three repetitions, the total duration of TI was significantly positively correlated with the time to first head movement in 11 of the 15 breeds. For the time to first leg movement, this correlation was even more pronounced as it was significantly positively correlated with the total duration of TI in 13 out of 15 breeds for the first repetition and 12 out of 15 breeds in the second and third repetition. In contrast, the number of attempts needed to induce TI was significantly and negatively correlated with the duration of TI in one out of 15 breeds in the first repetition, none in the second and one out of 15 in the third repetition (Table S4).

The consistency among individuals was *W* = 0.045 for the total duration of TI (*N* = 117, *χ*^2^ = 10.609, *df* = 2, *p* = 0.005; all other *p* ≥ 0.05). The consistency within individuals for the number of attempts was *W* = 0.559 (*N* = 3, *χ*^2^ = 194.391, *df* = 116, *p* ≤ 0.001), for the first head movement *W* = 0.647 (*N* = 3, *χ*^2^ = 225.192, *df* = 116, *p* ≤ 0.001), for the first leg movement *W* = 0.604 (*N* = 3, *χ*^2^ = 210.157, *df* = 116, *p* ≤ 0.001), and for the total duration of TI W = 0.630 (*N* = 3, *χ*^2^ = 219.160, *df* = 116, *p* ≤ 0.001).

## Discussion

### Breed-specific variation

The number of attempts needed to induce TI was not sensitive to breed variability in the initial repetition, whether or not they were organized into the categories of the breeds’ intended use. The threshold to induce TI was similar for all naïve chickens tested, irrespective of breed. The number of attempts ranged from 1 to 12, with most showing the induction of TI with the first attempt. These findings contrast with the recent literature comparing different breeds. Nelson et al. (2020) reported differences in attempts needed to induce TI among layer breeds (means of 1.4 for Rhode Island Red and White Plymouth Rock, and 1.8 for White Leghorn) and Yoshidome et al. (2021) reported differences among native Japanese chicken breeds (median of 1.5 for Tosa-Kukin, and 2.7 for Yakido chicken). Whereas the Japanese chickens were tested early at an age of 5 days (Yoshidome et al., 2021), the layer breeds Nelson et al. (2020) used were tested at an older age of 63 weeks. In our study, the animal’s age did not have an impact on the attempts needed to induce TI, although only mature birds were used. Assuming age did not have a significant impact, there are other factors that could have influenced the results, including rearing conditions and experiences, and handling of the animals. Whether handling has an impact on TI measurements is unclear. According to Kujiyat, Craig & Dayton (1983) handling does not affect TI measurements, whereas Jones (1992) showed that rough handling prolonged the duration of TI. The chickens used in this study were handled regularly and carefully and were therefore easy to catch, minimizing distress for the bird and impact on the TI measurements.

It is also unclear if the position within the pecking order had an effect on the individual values was as this was not assessed during the study. Crawford (1977) and Jones & Faure (1982) found contradicting results in the correlation of TI duration and dominance. This information could, in future studies, be accounted for through observation and/or by using replications of pens in the study. This, unfortunately, was not possible in this specific approach based on the variety of different native breeds.

In contrast to testing in naïve birds, the number of attempts needed to induce TI were affected by breed in Part B of the study, when looking at inducing TI over multiple repetitions. Here, the Ohiki stand out with a mean value of almost 5 attempts needed to induce TI. This finding could be linked to breed-specific differences in habituation towards TI, although we cannot support this idea further based on our statistics and sample sizes. This idea would be in line with findings from Abe et al. (2013) who also found White Leghorn chicks to show shorter TI durations initially, with a gradual increase during repeated testing. When they tested the Japanese dual-purpose Nagoya chicks, the birds showed initially longer TI durations with a gradual decrease over repetitions, suggesting habituation to the TI testing. The ability to adapt enables animals to better cope with environmental challenges, resulting in a higher welfare status (Arndt, Goerlich & van der Staay, 2022), and making the rate of habituation of high interest for future research.

The duration of TI was breed sensitive. Duration measures, including time to first head movement, first leg movement and total duration of TI, differed among breeds, but were not affected by age. In the majority of the breeds, these three time-related variables were correlated. This correlation on the breed level leads to the assumption that measuring one variable, preferably total duration of TI, might be sufficient for future studies. There were some breeds, however, in which these three variables were not correlated. This could either be due to small sample sizes or might be impacted by other factors. As there have been findings on longer latencies of head movements in males (Jones & Faure, 1981a) and in dominant hens (Jones & Faure, 1982), further research on larger sample sizes should address the question of correlation between these variables.

The majority of studies have focused on total TI duration. In our study, total durations ranged from 12 s (close to the 10 s minimal duration for TI induction) to 600 s (our maximal duration), with most in the range of 243 s to 321 s. The variation in the duration of TI reflects the variation among breeds tested for the TI response. The median TI duration of German Creeper was 10 times longer than that of the Lohmann Brown. German Creeper belong to the category of ornamental breeds which also includes Berg. Long Crower and East Frisian Gull, all of which had prolonged TI durations. Among the German Creepers, most of the TI measurements had to be artificially terminated at the maximum of 600 s, reflecting a strong fear response.

We were unable to find published data on the TI responses for most of the breeds used in this study. Only a few studies used similar breeds *e.g.*, Schütz, Forkman & Jensen, 2001; Schütz et al., 2004). The white Leghorn from Schütz, Forkman & Jensen (2001) showed a TI duration of 114 s, comparable to our white Leghorn strain LSL Classic which showed a TI duration of 98 s. In most of the studies, unfortunately, the exact breed is not stated for example in Nelson et al. (2020) who report three white Leghorn strains with an overall mean of 299 s. The name Leghorn is used interchangeably for hybrid lines and distinct breeds, such as the Italian Leghorn which is the ancestor to modern layer “Leghorn” lines. The most comparable data for the Italian Leghorn might be found in a study from Castellini et al. (2016) who report 38 s for TI duration in this breed as compared to 234 s in our study. These differences might either be linked to methodological differences or could indicate differences in breeding, and therefore the genetic background, of Italian and German Leghorns. Different populations might still carry the same breed’s name, but have different genetic backgrounds due to breeding history comparable to allopatric evolution in the wild. With focus on the phenotyping of chicken breeds, specific information should be given on the population (breed or hybrid line, or at least country of breeding population) as well as the duration of TI itself.

Typically, studies on tonic immobility and fear in chickens are focused on commercial lines (Dudde et al., 2018). There are only a few studies integrating other native or indigenous breeds, such as Indian Vanaraja (Panigrahy et al., 2017), English Ixworth (Albentosa, Kjær & Nicol, 2003), Spanish (Campo & Redondo, 1996), and red jungle fowl (Garnham & Løvlie, 2018). These previous studies all found large effects of breed on the probability of TI induction and the duration of the TI response. Moroi et al. (2019) applied a handling test, but not a TI test, to different Japanese breeds, including both dual-purpose and egg laying breeds. Dual-purpose chickens, Rhode Island Red, Australorp and Nagoya, were touchable in the test, which was regarded as passive tameness, whereas the other ornamental chickens avoided human contact. Applying this test to the breeds examined in this study would have been an additional parameter indicating fear-related behaviour towards humans.

There is a special interest, due to current ethical concerns and upcoming legal regulations on the killing of day-old male chicks in layer lines, on dual-purpose chickens (*e.g.*, Germany starting 2022; Tiemann, Hillemacher & Wittmann, 2020; Haas, Oliemans & van Gerwen, 2021). In general, the expectations of dual-purpose chickens solving potential welfare problems of high performing hybrids are high. Some studies show lowering the bird performance can improve welfare (Giersberg, Spindler & Kemper, 2020; Dawson et al., 2021), but additional validation, including fear, is needed (Mellor & Beausoleil, 2015). The dual-purpose native breeds tested in this study showed comparable TI durations with intermediate values, between hybrids and ornamental breeds.

The hybrid lines that we tested could be divided into layer-type lines with low TI durations and dual-purpose and meat-type lines with prolonged TI durations. The highly inbred layer hybrid lines, Lohmann Brown and LSL Classic had the shortest TI durations in the study, whereas median TI durations for the recently established hybrid line Lohmann Dual (dual-purpose line with dwarf factor) and the Cobb 500 breeder (parental line of high performing broiler) are in the upper third of the breeds tested. A previous study of Lohmann Brown (Johnsen, Vestergaard & Nørgaard-Nielsen, 1998) found much longer TI durations (approximately 300 s compared to 58 s here). These differences could be due to the ongoing selection process, especially in the high performing (and highly selected) commercial hybrid lines. There have been almost 25 generations of selection on Lohmann Brown between Johnsen, Vestergaard & Nørgaard-Nielsen (1998) and our study. It could be assumed that selection, especially towards alternative housing systems could have altered fear response, habituation and/or adaptation. This selection would result in shorter TI durations and could, therefore, be an explanation for the differences found. A study of LSL Classic (Wei et al., 2020) also reported much longer TI durations than we found (approximately 338 s compared to 98 s here). This difference may have been related to the housing system, *e.g.*, the use of furnished cages in the study of Wei et al. (2020) *vs.* free range in ours, as there are indications that free-range housing could also correlate with TI response, although the quality of that correlation remains unclear (Larsen et al., 2018). We found prolonged TI durations in Lohmann Dual, indicating a high fear response, although this breed has been reported to show low fear responses in other tests (Giersberg, Spindler & Kemper, 2020). This discrepancy might be due to the tests applied, TI in our study, but avoidance distance and novel object test in the study of Giersberg et al. (2020). It would be of interest to apply a test sequence of different standardized methods to a larger group of individuals in order to find correlations across studies, across tests, and among individual test outcomes.

The impact of morphological traits on the outcome of TI testing should be examined, although we did not find a clear pattern (*e.g.*, TI durations of short-legged German creeper and short-legged Japanese bantam were opposed). A correlation between morphology and TI test results has been reported for the red-winged tinamou, *Rhynchotus rufescens*, with shorter TI duration found for animals with greater wing length (Oliveira Santos et al., 2020). The inclusion of the morphological measures, including wing length, might therefore contribute to a more explicit interpretation of the TI response in future studies.

### Correlation of attempts to induce TI and duration

A correlation between the attempts needed to induce TI and the total duration of TI was found in only 3 of the 19 breeds in the first test. The low correlation may be influenced by the small sample size. The *post-hoc* power analysis indicated that sample sizes were too low to detect significant effects in some of the breeds (seven out of 19), so the correlations in these breeds are not robust. On the other hand, the lack of an association between these two variables supports models from Edelaar et al. (2012) reflecting that attempts needed and TI duration are controlled by different mechanisms. Among those studies that examined the correlation of the two variables, Abe et al. (2013) did not find a significant correlation within the combined data set of all 144 chickens of two breeds, the white Leghorn and Nagoya Cochin chickens, although they did find a significant positive correlation in one of three Nagoya strains. Ferrante et al. (2016) found a low negative correlation between the number of attempts needed to induce TI and the duration of TI for only one of the three local Italian breeds. Interestingly, Pittet et al. (2019) found a positive correlation between both variables in chicks raised by hens, but not in artificially reared chicks. The latter results might therefore correspond to those of the present study where all tested chickens were reared artificially. It would be of interest to determine whether the correlation between attempts to induce and duration of TI would change under different rearing conditions.

Our results which did show a low negative correlation of the number of attempts and TI duration are in line with studies on quail selected for different TI durations (Hazard et al., 2008). Quail selected for a short duration of TI showed a greater increase of attempts needed to induce TI during repeated testing than quails selected for long TI duration. This individual pattern of TI response during repeated testing is of high interest as it reflects possible personality traits and coping strategies that could be linked to a reduction in fear susceptibility, and improved adaptation and animal welfare.

### Clustering of breed categories

With the goal of achieving higher welfare levels, researchers, as well as farmers, are looking for alternative breeds in poultry production (Franzoni et al., 2021). Whether the use of dual-purpose chickens will improve animal welfare in poultry is examined in recent studies, but only a few take the animals’ emotional state into account (Meuser et al., 2021). Knowing whether breeds’ responses cluster according to their intended use, and how pronounced the fear response is, helps in deciding which breed categories would potentially be useful in lowering distress and improving welfare.

We found that breed categories range along a continuum of TI durations, with layer-type native breeds and especially ornamental breeds showing longer TI durations, and bantam, dual-purpose and meat-type native breeds showing intermediate TI durations. Commercial hybrids showed shortest TI duration, with layers ranging among the breeds with shorter TI, whereas dual-purpose and broiler breeder hybrids range among the breeds with longer TI durations.

The establishment of the categories reflecting the intended use of the breeds was done to discuss fear responses on a scale level above the single breed. Not all categories contained as many individuals and breeds as the others. Meat-type native chickens were especially underrepresented in the sample population, but this category did not substantially differ from the other categories in their TI response. In contrast, commercial hybrid lines, as well as bantam and ornamental native breeds did all cover a similar number of individuals but were found to differ from each other in their TI duration. Still, the number of breeds differs between categories and might have caused a bias, and increasing sample sizes of underrepresented categories might change the outcome. Other studies categorized chicken breeds based on their genetic relationship and geographical origin (Malomane et al., 2019) or domestic dog breeds according to their genetic relatedness, behaviour and trainability (Turcsán, Kubinyi & Miklósi, 2011). The dog study mentioned the contribution to the welfare if dog (categories) and owner (expectation) match. Transferring this approach, the matching of farmers/farm environments and chicken (categories) should also support animal welfare. The knowledge of the typical character of a breed category is advantageous and should be taken into consideration in future research as well as in the selection of production birds.

Although, breeds (and the corresponding individuals) were assigned to the categories based on their intended use, we found differences between categories based on TI responses. Hybrid lines were found showing short, bantam, meat-type and dual-purpose native breeds intermediate, and layer-type and ornamental native breeds longer TI durations. This overview of the TI responses at least provides a possible assessment of which breeds show the lowest fear response and could therefore be recommended for use.

### Limitations and future prospects

The limitation and distinctive feature of this study is the wide range of breeds tested. Unfortunately, scientific literature or studies on native breeds including intended use, breeding history and goal, or predominantly applied husbandry systems, are rare. Also, comparable TI data for the breeds included in our study are missing.

Reliability was shown to be high based on the assumptions of consistency testing in this study. In general, standardization of testing methods (*e.g.*, SHIRPA, Rogers et al., 1997), and describing the research set-up and procedure in which the animals are observed (Murphy, Nordquist & van der Staay, 2014) would improve the comparability across studies. Still, our results might be affected by low sample sizes within breeds and high variability among individuals at the same time. The limited availability of animal genetic resources required the use of birds with different rearing experiences and of different ages to be tested, and resulted in a lack of replications among others. Therefore, rearing environment as well as the limitation of housing one flock per breed might impact our data. A more strategic approach might therefore need facilities that offer hatching, rearing and housing of larger numbers of individuals to improve reliability. Chickens are raised in a wide variety of environments, however, and future studies examining fear should represent this variety.

The breed differences found might also be associated with other behavioural traits affecting fear response. For example, high locomotory activity can account for high fear responses (Krause et al., 2019; Iffland et al., 2020). This might also reflect breed-specific differences in this study, although comparative values or measurements of locomotory activity of the breeds are lacking. Influences such as age (Hocking et al., 2001), feeding management (Kraimi et al., 2019), handling before testing (Duncan et al., 1986), calls of other chickens (Pochron & Thompson, 2019), husbandry, and litter (Hocking, Jones & Picard, 2005; Brantsæter et al., 2017), were controlled, or if not possible (rearing history including time of placement at the research station), balanced among the animals tested. Age, in particular, was included in the statistical model, but, in contrast to a previous study (Hocking et al., 2001), we found no effect. The consistency between animals has been found to reflect the individual quantitative variation. Notably, the consistency within individuals among the parameters measured ranged between 0.56 and 0.65, reflecting the reliability of the dataset for duration measures. Consistency for the number of attempts to induce TI should be taken with caution due to the limited variation, with most chickens requiring 1 attempt to induce TI.

### Repeated testing

Whereas TI duration differed between breeds in both parts of the study, the number of attempts needed to induce TI was not impacted by breed among naïve chickens whereas it differed between breeds during repeated testing.

Although, the median number of attempts was constant at one attempt to induce TI, the range increased to a maximum of 22 attempts in the 2nd repetition and to 38 attempts in the 3rd repetition. Interestingly, these few high values were gained from the same individuals across repetitions, two Ohiki and two Polands. This finding is also reflected in the consistency testing showing high repeatability in the same animal. In addition, this finding is in line with the lack of correlation between number of attempts to induce TI and the total duration of TI as the mentioned two Ohiki and two Poland hens did not show shorter TI durations compared to the other tested animals. The differences in fear susceptibility between breeds could be of interest for future research on emotions, personality traits, and coping strategies (Janczak et al., 2003; Carter et al., 2013; Pusch & Navara, 2018; Kozak et al., 2019; Krause et al., 2019).

Repeated testing might enhance breed-specific differences. In general, animals with a low fear response are less susceptible to inducing TI, and this is enhanced through repeated testing (Ghareeb et al., 2008). Adaptation might enable the individual to experience less fear and achieve a higher welfare level. In turn, TI testing is a reliable procedure for testing genetic predisposition for TI response and genetically based fear susceptibility in general based on the high repeatability of this variable (Hocking et al., 2001; Fogelholm et al., 2019).

A study by Nash & Gallup (1976) found habituation effects during TI testing in chickens, whereas Okpokho & Craig (1987) reported that the duration of TI did not change with habituation. Whether habitation affects TI response or not, might be a question of the experimental design. With a different species, domestic rabbits, Smith & Klemm (1977) did not find habituation effects, although TI was applied more than 750 times in one of the rabbits. Inconsistent findings for habituation, also stated by Jones (1986), might be explained by a study on sharks showing that habituation appears within a daily session, but TI durations tested on different days might show comparable length (Watsky & Gruber, 1990). We did not find a significant habituation effect, but this might be due to procedural limitations. Ensuring the welfare of the animals, our daily limit was three inductions, no matter whether TI was successfully induced or not. This resulted in approximately half of the chickens being tested in one day, and the other half receiving sessions on multiple successive days until TI was successfully induced three times. Future studies on habituation of the TI response should therefore follow a more rigorous experimental design that pays attention to the possible different habituation effects on the number of attempts and the total duration of TI.

Our results support the hypothesis that selection has altered chicken behaviour, resulting in breed-specific fear responses and emotional reactivity. Based on the variety of chicken breeds and their TI responses, breeding for TI (Remignon et al., 1998; Hazard et al., 2008) and the ability for habituation and adaptation might favour high standards of animal welfare based on the animal’s capacity. The basic message of the studies is the same: selection can and should reduce fear, favour adaptation (including habituation) and increase animal welfare (Meuser et al., 2021). Recently, Ferreira, Guesdon & Calandreau (2021) proposed adjusting environment to the chicken’s behavioural capabilities, enabling the animal to cope, interact and adapt to the individual’s environment, resulting in an improvement in welfare. Here, we show the variation of TI responses in the domestic chicken. This variation could be used as a template for future breeding towards improved animal welfare.

## Conclusions

This study aimed to assess the diversity of chickens and their reaction in a fear-evoking standardized test, the tonic immobility test. We found breed-specific differences in the fear response. Our results might contribute to the description of fear susceptibility of breeds and breed categories, and whether habituation mechanisms could be found among breeds. To gain more insight into the individual predisposition for experiencing fear, future research should emphasize the observation of individuals with controlled life-histories. The goal should be to examine possible ways of breeding for behavioural predispositions and responses that favour the ability of the individual to cope with and adapt to demanding environmental factors.

##  Supplemental Information

10.7717/peerj.14703/supp-1 Supplemental Information 1 TI response parameters part A The analysis of the Tonic immobility (TI) response of naïve, mature hens of different breeds (part A) is given with the fixed effect breed and random effect age. *p*-values are marked with * for significances ( *α*-level was set at *p* ≤ 0.05 and indicated as *, *p* ≤ 0.01 is indicated as ** and *p* ≤ 0.001 as ***). Only significant pairwise comparisons (*p* ≤ 0.05) are highlighted green.

10.7717/peerj.14703/supp-2 Supplemental Information 2 Correlations in part A The analysis of the Tonic immobility (TI) response of naïve, mature hens of different breeds (part A) is given with the coefficients breed and age. Reported are the results for all measured TI response parameters. *p*-values are marked with * for significances ( *α*-level was set at *p* ≤ 0.05 and indicated as *, *p* ≤ 0.01 is indicated as ** and *p* ≤ 0.001 as ***). Only significant pairwise comparisons (*p* ≤ 0.05) are highlighted green.

10.7717/peerj.14703/supp-3 Supplemental Information 3 TI response parameters part B The analysis of the Tonic immobility (TI) response of mature hens of different breeds during repeated testing (part B) is given with the fixed effect breed and random effect age. *p*-values are marked with * for significances ( *α*-level was set at *p* ≤ 0.05 and indicated as *, *p* ≤ 0.01 is indicated as ** and *p* ≤ 0.001 as ***). Only significant pairwise comparisons (*p* ≤ 0.05) are highlighted green.

10.7717/peerj.14703/supp-4 Supplemental Information 4 Correlations in part B The analysis of the Tonic immobility (TI) response of mature hens of different breeds during repeated testing (part B) is given with the coefficients breed and age. Reported are the results for all measured TI response parameters.*p*-values are marked with * for significances ( *α*-level was set at *p* ≤ 0.05 and indicated as *, *p* ≤ 0.01 is indicated as ** and *p* ≤ 0.001 as ***). Only significant pairwise comparisons (*p* ≤ 0.05) are highlighted green.

10.7717/peerj.14703/supp-5 Supplemental Information 5 The power analysis A power analysis has been conducted on the complete data set to indicate sufficient sample sizes or underrepresented chicken breeds.

10.7717/peerj.14703/supp-6 Supplemental Information 6 Raw data of the TI responses All raw data of the study are provided for part A (naive chickens) as well as for part B (repeated testing of chickens).

## Additional Information and Declarations

### Data Availability

The following information was supplied regarding data availability: The raw data and results are available in the Supplementary Tables.
