# Supplementary material for: Differences among domestic chicken breeds in tonic immobility responses as a measure of fearfulness
Source: PeerJ. 2023 Apr 4;11:e14703. doi: 10.7717/peerj.14703 (PMC10081456; doi:10.7717/peerj.14703)
Supplement: Supplemental Information 1 — The analysis of the Tonic immobility (TI) response of naïve, mature hens of different breeds (part A) is given with the fixed effect breed and random effect age. p-values are marked with * for significances ( α-level was set at p ≤ 0.05 and indicated as *, p ≤ 0.01 is indicated as ** and p ≤ 0.001 as ***). Only significant pairwise comparisons (p ≤ 0.05) are highlighted green. [file peerj-11-14703-s001.docx]

**Supplemental table S1**

Part A:

**1^st^ Head movement x Breed**

## Holm-Bonferroni post-hoc tests for pairwise comparisons of breeds for the variable first head movement of naïve, mature hens of different breeds (part A). Given are the estimate. which indicates the difference between the pair (first minus second), standard error (SE) and p-value. P-values are marked with * for significances (α-level was set at p ≤ 0.05 and indicated as *, p ≤ 0.01 is indicated as ** and p ≤ 0.001 as ***). Only significant pairwise comparisons (p ≤ 0.05) are highlighted green.

| **Breeds** | **Estimate** | **SE** | **p-value** |
| --- | --- | --- | --- |
| SI - BLC | -5.873 | 3.480 | 0.973 |
| SI - BS | -1.496 | 3.090 | 1.000 |
| SI - BR | -0.662 | 3.480 | 1.000 |
| SI - CB | -5.861 | 3.900 | 0.992 |
| SI - CO | 1.785 | 3.230 | 1.000 |
| SI - EFG | -1.260 | 3.720 | 1.000 |
| SI - GC | -8.537 | 3.330 | 0.512 |
| SI - GEB | 0.420 | 3.370 | 1.000 |
| SI - JB | 1.892 | 3.080 | 1.000 |
| SI - LG | 5.259 | 3.720 | 0.996 |
| SI - LB | 10.330 | 3.370 | 0.200 |
| SI - LD | 1.242 | 3.630 | 1.000 |
| SI - LSL | 5.486 | 3.110 | 0.959 |
| SI - MA | -1.484 | 3.900 | 1.000 |
| SI - OH | 3.156 | 3.400 | 1.000 |
| SI - PO | -3.521 | 3.200 | 1.000 |
| SI - RB | 4.339 | 4.160 | 1.000 |
| SI - YO | -3.600 | 4.050 | 1.000 |
| BLC - BS | 4.377 | 2.920 | 0.992 |
| BLC - BR | 5.211 | 3.210 | 0.982 |
| BLC - CB | 0.012 | 3.710 | 1.000 |
| BLC - CO | 7.658 | 2.900 | 0.458 |
| BLC - EFG | 4.613 | 3.510 | 0.998 |
| BLC - GC | -2.664 | 3.100 | 1.000 |
| BLC - GEB | 6.292 | 3.250 | 0.908 |
| BLC - JB | 7.765 | 2.790 | 0.358 |
| BLC - LG | 11.132 | 3.510 | 0.155 |
| BLC - LB | 16.203 | 3.250 | 0.000 |
| BLC - LD | 7.114 | 3.510 | 0.870 |
| BLC - LSL | 11.359 | 2.930 | 0.020 |
| BLC - MA | 4.388 | 3.670 | 1.000 |
| BLC - OH | 9.028 | 3.150 | 0.302 |
| BLC - PO | 2.351 | 2.830 | 1.000 |
| BLC - RB | 10.211 | 3.560 | 0.303 |
| BLC - YO | 2.272 | 3.490 | 1.000 |
| BS - BR | 0.834 | 2.810 | 1.000 |
| BS - CB | -4.365 | 3.290 | 0.998 |
| BS - CO | 3.280 | 2.510 | 0.999 |
| BS - EFG | 0.236 | 3.070 | 1.000 |
| BS - GC | -7.041 | 2.590 | 0.397 |
| BS - GEB | 1.915 | 2.570 | 1.000 |
| BS - JB | 3.388 | 2.290 | 0.993 |
| BS - LG | 6.755 | 3.070 | 0.776 |
| BS - LB | 11.826 | 2.570 | 0.001 |
| BS - LD | 2.737 | 2.900 | 1.000 |
| BS - LSL | 6.982 | 2.240 | 0.178 |
| BS - MA | 0.011 | 3.310 | 1.000 |
| BS - OH | 4.651 | 2.680 | 0.965 |
| BS - PO | -2.026 | 2.490 | 1.000 |
| BS - RB | 5.834 | 3.820 | 0.990 |
| BS - YO | -2.105 | 3.680 | 1.000 |
| BR - CB | -5.199 | 3.680 | 0.996 |
| BR - CO | 2.447 | 2.940 | 1.000 |
| BR - EFG | -0.598 | 3.480 | 1.000 |
| BR - GC | -7.875 | 3.060 | 0.506 |
| BR - GEB | 1.082 | 3.120 | 1.000 |
| BR - JB | 2.554 | 2.790 | 1.000 |
| BR - LG | 5.921 | 3.480 | 0.971 |
| BR - LB | 10.992 | 3.120 | 0.059 |
| BR - LD | 1.903 | 3.390 | 1.000 |
| BR - LSL | 6.148 | 2.830 | 0.790 |
| BR - MA | -0.823 | 3.670 | 1.000 |
| BR - OH | 3.817 | 3.130 | 0.999 |
| BR - PO | -2.860 | 2.910 | 1.000 |
| BR - RB | 5.001 | 3.910 | 0.999 |
| BR - YO | -2.938 | 3.810 | 1.000 |
| CB - CO | 7.646 | 3.440 | 0.761 |
| CB - EFG | 4.601 | 3.900 | 1.000 |
| CB - GC | -2.676 | 3.530 | 1.000 |
| CB - GEB | 6.281 | 3.550 | 0.958 |
| CB - JB | 7.753 | 3.300 | 0.674 |
| CB - LG | 11.120 | 3.900 | 0.313 |
| CB - LB | 16.191 | 3.560 | 0.002 |
| CB - LD | 7.102 | 3.800 | 0.931 |
| CB - LSL | 11.347 | 3.310 | 0.078 |
| CB - MA | 4.377 | 4.080 | 1.000 |
| CB - OH | 9.017 | 3.590 | 0.554 |
| CB - PO | 2.340 | 3.420 | 1.000 |
| CB - RB | 10.200 | 4.370 | 0.685 |
| CB - YO | 2.261 | 4.270 | 1.000 |
| CO - EFG | -3.045 | 3.240 | 1.000 |
| CO - GC | -10.322 | 2.780 | 0.032 |
| CO - GEB | -1.365 | 2.860 | 1.000 |
| CO - JB | 0.107 | 2.470 | 1.000 |
| CO - LG | 3.474 | 3.240 | 1.000 |
| CO - LB | 8.545 | 2.860 | 0.235 |
| CO - LD | -0.543 | 3.150 | 1.000 |
| CO - LSL | 3.701 | 2.530 | 0.994 |
| CO - MA | -3.269 | 3.430 | 1.000 |
| CO - OH | 1.371 | 2.850 | 1.000 |
| CO - PO | -5.306 | 2.580 | 0.857 |
| CO - RB | 2.554 | 3.630 | 1.000 |
| CO - YO | -5.385 | 3.520 | 0.990 |
| EFG - GC | -7.277 | 3.330 | 0.782 |
| EFG - GEB | 1.680 | 3.350 | 1.000 |
| EFG - JB | 3.152 | 3.090 | 1.000 |
| EFG - LG | 6.519 | 3.720 | 0.961 |
| EFG - LB | 11.590 | 3.360 | 0.072 |
| EFG - LD | 2.501 | 3.610 | 1.000 |
| EFG - LSL | 6.746 | 3.100 | 0.788 |
| EFG - MA | -0.225 | 3.900 | 1.000 |
| EFG - OH | 4.415 | 3.400 | 0.999 |
| EFG - PO | -2.262 | 3.210 | 1.000 |
| EFG - RB | 5.599 | 4.210 | 0.998 |
| EFG - YO | -2.340 | 4.100 | 1.000 |
| GC - GEB | 8.957 | 2.910 | 0.194 |
| GC - JB | 10.429 | 2.600 | 0.012 |
| GC - LG | 13.796 | 3.330 | 0.007 |
| GC - LB | 18.867 | 2.910 | ≤ 0.001*** |
| GC - LD | 9.778 | 3.200 | 0.204 |
| GC - LSL | 14.023 | 2.610 | ≤ 0.001*** |
| GC - MA | 7.053 | 3.530 | 0.884 |
| GC - OH | 11.693 | 2.960 | 0.015 |
| GC - PO | 5.016 | 2.750 | 0.944 |
| GC - RB | 12.876 | 3.870 | 0.103 |
| GC - YO | 4.937 | 3.750 | 0.998 |
| GEB - JB | 1.473 | 2.660 | 1.000 |
| GEB - LG | 4.840 | 3.350 | 0.995 |
| GEB - LB | 9.910 | 2.880 | 0.075 |
| GEB - LD | 0.822 | 3.170 | 1.000 |
| GEB - LSL | 5.067 | 2.600 | 0.904 |
| GEB - MA | -1.904 | 3.580 | 1.000 |
| GEB - OH | 2.736 | 3.000 | 1.000 |
| GEB - PO | -3.941 | 2.850 | 0.997 |
| GEB - RB | 3.919 | 4.110 | 1.000 |
| GEB - YO | -4.020 | 3.970 | 1.000 |
| JB - LG | 3.367 | 3.090 | 1.000 |
| JB - LB | 8.438 | 2.660 | 0.154 |
| JB - LD | -0.651 | 2.970 | 1.000 |
| JB - LSL | 3.594 | 2.320 | 0.989 |
| JB - MA | -3.377 | 3.300 | 1.000 |
| JB - OH | 1.264 | 2.680 | 1.000 |
| JB - PO | -5.414 | 2.430 | 0.754 |
| JB - RB | 2.447 | 3.590 | 1.000 |
| JB - YO | -5.492 | 3.470 | 0.986 |
| LG - LB | 5.071 | 3.360 | 0.992 |
| LG - LD | -4.018 | 3.610 | 1.000 |
| LG - LSL | 0.227 | 3.100 | 1.000 |
| LG - MA | -6.744 | 3.900 | 0.966 |
| LG - OH | -2.104 | 3.400 | 1.000 |
| LG - PO | -8.781 | 3.210 | 0.390 |
| LG - RB | -0.920 | 4.210 | 1.000 |
| LG - YO | -8.860 | 4.100 | 0.799 |
| LB - LD | -9.089 | 3.170 | 0.306 |
| LB - LSL | -4.844 | 2.600 | 0.934 |
| LB - MA | -11.814 | 3.580 | 0.110 |
| LB - OH | -7.174 | 3.010 | 0.645 |
| LB - PO | -13.851 | 2.860 | 0.001 |
| LB - RB | -5.991 | 4.120 | 0.994 |
| LB - YO | -13.930 | 3.970 | 0.062 |
| LD - LSL | 4.245 | 2.920 | 0.995 |
| LD - MA | -2.726 | 3.820 | 1.000 |
| LD - OH | 1.914 | 3.290 | 1.000 |
| LD - PO | -4.763 | 3.150 | 0.991 |
| LD - RB | 3.097 | 4.320 | 1.000 |
| LD - YO | -4.842 | 4.180 | 1.000 |
| LSL - MA | -6.971 | 3.330 | 0.836 |
| LSL - OH | -2.331 | 2.710 | 1.000 |
| LSL - PO | -9.008 | 2.510 | 0.049 |
| LSL - RB | -1.148 | 3.820 | 1.000 |
| LSL - YO | -9.087 | 3.680 | 0.584 |
| MA - OH | 4.640 | 3.590 | 0.999 |
| MA - PO | -2.037 | 3.400 | 1.000 |
| MA - RB | 5.823 | 4.310 | 0.998 |
| MA - YO | -2.116 | 4.210 | 1.000 |
| OH - PO | -6.677 | 2.820 | 0.658 |
| OH - RB | 1.183 | 3.890 | 1.000 |
| OH - YO | -6.756 | 3.770 | 0.953 |
| PO - RB | 7.860 | 3.530 | 0.755 |
| PO - YO | -0.079 | 3.430 | 1.000 |
| RB - YO | -7.939 | 3.730 | 0.816 |

**1^st^ Leg movement x Breed**

Holm-Bonferroni post-hoc tests for pairwise comparisons of breeds for the variable **first leg movement** of naïve, mature hens of different breeds (part A). Given are the estimate, which indicates the difference between the pair (first minus second), standard error (SE) and p-value. P-values are marked with * for significances (α-level was set at p ≤ 0.05 and indicated as *, p ≤ 0.01 is indicated as ** and p ≤ 0.001 as ***). Only significant pairwise comparisons (p ≤ 0.05) are highlighted green.

| **Breeds** | **Estimate** | **SE** | **p - value** |
| --- | --- | --- | --- |
| SI - BLC | -4.836 | 3.170 | 0.991 |
| SI - BS | -0.054 | 2.770 | 1.000 |
| SI - BR | -0.193 | 3.160 | 1.000 |
| SI - CB | -5.042 | 3.580 | 0.996 |
| SI - CO | 1.650 | 2.910 | 1.000 |
| SI - EFG | -1.740 | 3.400 | 1.000 |
| SI - GC | -8.740 | 3.010 | 0.283 |
| SI - GEB | 1.388 | 3.050 | 1.000 |
| SI - JB | 2.302 | 2.770 | 1.000 |
| SI - LG | 3.117 | 3.400 | 1.000 |
| SI - LB | 7.861 | 3.060 | 0.506 |
| SI - LD | -0.725 | 3.300 | 1.000 |
| SI - LSL | 4.470 | 2.790 | 0.984 |
| SI - MA | -0.825 | 3.580 | 1.000 |
| SI - OH | 4.069 | 3.080 | 0.998 |
| SI - PO | -4.195 | 2.880 | 0.995 |
| SI - RB | 2.411 | 3.840 | 1.000 |
| SI - YO | -3.521 | 3.730 | 1.000 |
| BLC - BS | 4.783 | 2.770 | 0.966 |
| BLC - BR | 4.643 | 3.050 | 0.991 |
| BLC - CB | -0.206 | 3.510 | 1.000 |
| BLC - CO | 6.487 | 2.750 | 0.668 |
| BLC - EFG | 3.097 | 3.330 | 1.000 |
| BLC - GC | -3.904 | 2.940 | 0.998 |
| BLC - GEB | 6.224 | 3.080 | 0.873 |
| BLC - JB | 7.138 | 2.650 | 0.416 |
| BLC - LG | 7.953 | 3.330 | 0.646 |
| BLC - LB | 12.697 | 3.090 | 0.008 |
| BLC - LD | 4.111 | 3.330 | 0.999 |
| BLC - LSL | 9.306 | 2.780 | 0.098 |
| BLC - MA | 4.011 | 3.480 | 1.000 |
| BLC - OH | 8.905 | 2.980 | 0.237 |
| BLC - PO | 0.641 | 2.680 | 1.000 |
| BLC - RB | 7.247 | 3.380 | 0.807 |
| BLC - YO | 1.315 | 3.310 | 1.000 |
| BS - BR | -0.139 | 2.660 | 1.000 |
| BS - CB | -4.989 | 3.120 | 0.985 |
| BS - CO | 1.704 | 2.380 | 1.000 |
| BS - EFG | -1.686 | 2.920 | 1.000 |
| BS - GC | -8.686 | 2.450 | 0.055 |
| BS - GEB | 1.442 | 2.440 | 1.000 |
| BS - JB | 2.356 | 2.170 | 1.000 |
| BS - LG | 3.171 | 2.920 | 1.000 |
| BS - LB | 7.914 | 2.440 | 0.127 |
| BS - LD | -0.671 | 2.750 | 1.000 |
| BS - LSL | 4.523 | 2.130 | 0.819 |
| BS - MA | -0.771 | 3.140 | 1.000 |
| BS - OH | 4.123 | 2.540 | 0.982 |
| BS - PO | -4.141 | 2.360 | 0.962 |
| BS - RB | 2.464 | 3.620 | 1.000 |
| BS - YO | -3.468 | 3.490 | 1.000 |
| BR - CB | -4.849 | 3.490 | 0.997 |
| BR - CO | 1.843 | 2.790 | 1.000 |
| BR - EFG | -1.547 | 3.300 | 1.000 |
| BR - GC | -8.547 | 2.900 | 0.259 |
| BR - GEB | 1.581 | 2.960 | 1.000 |
| BR - JB | 2.495 | 2.650 | 1.000 |
| BR - LG | 3.310 | 3.300 | 1.000 |
| BR - LB | 8.054 | 2.960 | 0.397 |
| BR - LD | -0.532 | 3.210 | 1.000 |
| BR - LSL | 4.663 | 2.680 | 0.964 |
| BR - MA | -0.632 | 3.480 | 1.000 |
| BR - OH | 4.262 | 2.970 | 0.995 |
| BR - PO | -4.002 | 2.760 | 0.995 |
| BR - RB | 2.604 | 3.710 | 1.000 |
| BR - YO | -3.328 | 3.610 | 1.000 |
| CB - CO | 6.692 | 3.260 | 0.859 |
| CB - EFG | 3.302 | 3.700 | 1.000 |
| CB - GC | -3.698 | 3.350 | 1.000 |
| CB - GEB | 6.430 | 3.370 | 0.919 |
| CB - JB | 7.344 | 3.130 | 0.676 |
| CB - LG | 8.159 | 3.700 | 0.771 |
| CB - LB | 12.903 | 3.370 | 0.023 |
| CB - LD | 4.317 | 3.600 | 1.000 |
| CB - LSL | 9.512 | 3.140 | 0.215 |
| CB - MA | 4.217 | 3.870 | 1.000 |
| CB - OH | 9.111 | 3.410 | 0.432 |
| CB - PO | 0.847 | 3.240 | 1.000 |
| CB - RB | 7.453 | 4.150 | 0.952 |
| CB - YO | 1.521 | 4.050 | 1.000 |
| CO - EFG | -3.390 | 3.070 | 1.000 |
| CO - GC | -10.390 | 2.630 | 0.015 |
| CO - GEB | -0.262 | 2.710 | 1.000 |
| CO - JB | 0.652 | 2.340 | 1.000 |
| CO - LG | 1.467 | 3.070 | 1.000 |
| CO - LB | 6.210 | 2.710 | 0.714 |
| CO - LD | -2.375 | 2.990 | 1.000 |
| CO - LSL | 2.819 | 2.400 | 1.000 |
| CO - MA | -2.475 | 3.260 | 1.000 |
| CO - OH | 2.419 | 2.700 | 1.000 |
| CO - PO | -5.845 | 2.450 | 0.646 |
| CO - RB | 0.761 | 3.440 | 1.000 |
| CO - YO | -5.171 | 3.340 | 0.989 |
| EFG - GC | -7.000 | 3.150 | 0.762 |
| EFG - GEB | 3.128 | 3.180 | 1.000 |
| EFG - JB | 4.042 | 2.930 | 0.997 |
| EFG - LG | 4.857 | 3.530 | 0.997 |
| EFG - LB | 9.600 | 3.180 | 0.221 |
| EFG - LD | 1.015 | 3.420 | 1.000 |
| EFG - LSL | 6.209 | 2.940 | 0.825 |
| EFG - MA | 0.915 | 3.700 | 1.000 |
| EFG - OH | 5.809 | 3.220 | 0.950 |
| EFG - PO | -2.455 | 3.050 | 1.000 |
| EFG - RB | 4.151 | 4.000 | 1.000 |
| EFG - YO | -1.781 | 3.890 | 1.000 |
| GC - GEB | 10.128 | 2.760 | 0.038 |
| GC - JB | 11.042 | 2.470 | 0.002 |
| GC - LG | 11.857 | 3.150 | 0.028 |
| GC - LB | 16.601 | 2.760 | <.0001 |
| GC - LD | 8.015 | 3.040 | 0.457 |
| GC - LSL | 13.210 | 2.480 | 0.000 |
| GC - MA | 7.915 | 3.350 | 0.662 |
| GC - OH | 12.809 | 2.810 | 0.002 |
| GC - PO | 4.545 | 2.610 | 0.963 |
| GC - RB | 11.151 | 3.670 | 0.211 |
| GC - YO | 5.219 | 3.560 | 0.994 |
| GEB - JB | 0.914 | 2.520 | 1.000 |
| GEB - LG | 1.729 | 3.180 | 1.000 |
| GEB - LB | 6.473 | 2.730 | 0.658 |
| GEB - LD | -2.113 | 3.010 | 1.000 |
| GEB - LSL | 3.082 | 2.470 | 0.999 |
| GEB - MA | -2.213 | 3.390 | 1.000 |
| GEB - OH | 2.681 | 2.850 | 1.000 |
| GEB - PO | -5.583 | 2.700 | 0.852 |
| GEB - RB | 1.023 | 3.900 | 1.000 |
| GEB - YO | -4.909 | 3.760 | 0.999 |
| JB - LG | 0.815 | 2.930 | 1.000 |
| JB - LB | 5.559 | 2.520 | 0.773 |
| JB - LD | -3.027 | 2.820 | 1.000 |
| JB - LSL | 2.168 | 2.200 | 1.000 |
| JB - MA | -3.127 | 3.130 | 1.000 |
| JB - OH | 1.767 | 2.550 | 1.000 |
| JB - PO | -6.497 | 2.300 | 0.330 |
| JB - RB | 0.109 | 3.410 | 1.000 |
| JB - YO | -5.823 | 3.290 | 0.958 |
| LG - LB | 4.744 | 3.180 | 0.993 |
| LG - LD | -3.842 | 3.420 | 1.000 |
| LG - LSL | 1.352 | 2.940 | 1.000 |
| LG - MA | -3.942 | 3.700 | 1.000 |
| LG - OH | 0.952 | 3.220 | 1.000 |
| LG - PO | -7.312 | 3.050 | 0.635 |
| LG - RB | -0.706 | 4.000 | 1.000 |
| LG - YO | -6.638 | 3.890 | 0.970 |
| LB - LD | -8.586 | 3.010 | 0.312 |
| LB - LSL | -3.391 | 2.470 | 0.997 |
| LB - MA | -8.686 | 3.390 | 0.515 |
| LB - OH | -3.792 | 2.850 | 0.998 |
| LB - PO | -12.056 | 2.710 | 0.002 |
| LB - RB | -5.450 | 3.900 | 0.997 |
| LB - YO | -11.382 | 3.770 | 0.219 |
| LD - LSL | 5.195 | 2.770 | 0.930 |
| LD - MA | -0.100 | 3.620 | 1.000 |
| LD - OH | 4.794 | 3.120 | 0.990 |
| LD - PO | -3.470 | 2.980 | 1.000 |
| LD - RB | 3.136 | 4.090 | 1.000 |
| LD - YO | -2.796 | 3.970 | 1.000 |
| LSL - MA | -5.295 | 3.160 | 0.975 |
| LSL - OH | -0.401 | 2.570 | 1.000 |
| LSL - PO | -8.665 | 2.380 | 0.042 |
| LSL - RB | -2.059 | 3.620 | 1.000 |
| LSL - YO | -7.991 | 3.490 | 0.715 |
| MA - OH | 4.894 | 3.410 | 0.995 |
| MA - PO | -3.370 | 3.230 | 1.000 |
| MA - RB | 3.236 | 4.090 | 1.000 |
| MA - YO | -2.696 | 3.990 | 1.000 |
| OH - PO | -8.264 | 2.670 | 0.186 |
| OH - RB | -1.658 | 3.690 | 1.000 |
| OH - YO | -7.590 | 3.580 | 0.822 |
| PO - RB | 6.606 | 3.340 | 0.893 |
| PO - YO | 0.674 | 3.250 | 1.000 |
| RB - YO | -5.932 | 3.530 | 0.975 |

**Duration of TI x Breed**

Pairwise comparisons (Holm-Bonferroni-adjusted) between breeds of naïve, mature hens (part A) concerning the duration of TI revealed no significant differences (all p > 0.05).

**Attempts to induce TI x Breed**

No pairwise comparisons were conducted as there was no significant impact of breed on the attempts needed to induce TI.
