# Supplementary material for: Differences among domestic chicken breeds in tonic immobility responses as a measure of fearfulness
Source: PeerJ. 2023 Apr 4;11:e14703. doi: 10.7717/peerj.14703 (PMC10081456; doi:10.7717/peerj.14703)
Supplement: Supplemental Information 2 — The analysis of the Tonic immobility (TI) response of naïve, mature hens of different breeds (part A) is given with the coefficients breed and age. Reported are the results for all measured TI response parameters. p-values are marked with * for significances ( α-level was set at p ≤ 0.05 and indicated as *, p ≤ 0.01 is indicated as ** and p ≤ 0.001 as ***). Only significant pairwise comparisons (p ≤ 0.05) are highlighted green. [file peerj-11-14703-s002.docx]

**Table S2:** Correlations between the first head movement, the first leg movement, the number of inductions and the total duration of tonic immobility (turn) within each breed. The sample size (N), the correlation coefficient (r) and the significance level (p) are given.

|  | **First Head Movement - Turn** | | | **First Leg Movement - Turn** | | | **Number of inductions - Turn** | | |
| --- | --- | --- | --- | --- | --- | --- | --- | --- | --- |
| **Breed** | N | r | p | N | r | p | N | r | p |
| Bantam Silkie | 6 | 0.943 | 0.005 | 7 | 0.964 | < 0.001 | 7 | 0.359 | 0.430 |
| Bergische Long Crower | 9 | 0.945 | < 0.001 | 9 | 0.945 | < 0.001 | 9 | -0.429 | 0.249 |
| Bergische Schlotterkämm | 17 | 0.663 | 0.004 | 17 | 0.526 | 0.030 | 17 | 0.358 | 0.158 |
| Breda | 8 | 0.994 | < 0.001 | 8 | 1 | ** | 8 | -0.415 | 0.307 |
| Cobb 500 | 5 | 1 | < 0.001 | 5 | 1 | < 0.001 | 5 | * | * |
| Cochin | 12 | 0.818 | 0.001 | 12 | 0.832 | < 0.001 | 12 | 0.218 | 0.495 |
| East Frisian Gull | 6 | 0.274 | 0.599 | 6 | 0.920 | 0.009 | 6 | -0.845 | 0.034 |
| German Creeper | 10 | 0.982 | < 0.001 | 10 | 1 | ** | 10 | * | * |
| German Empire Breed | 10 | 0.867 | 0.001 | 10 | 0.955 | < 0.001 | 10 | 0.725 | 0.018 |
| Japanese bantam | 16 | 0.846 | < 0.001 | 16 | 0.893 | < 0.001 | 16 | -0.224 | 0.404 |
| Leghorn | 6 | 0.600 | 0.208 | 6 | 0.429 | 0.397 | 6 | -0.169 | 0.749 |
| Lohmann Brown | 10 | 0.395 | 0.258 | 10 | 0.665 | 0.036 | 10 | -0.174 | 0.631 |
| Lohmann Dual | 7 | 0.296 | 0.518 | 7 | 0.786 | 0.036 | 7 | 0.00 | 1.00 |
| LSL | 16 | 0.439 | 0.089 | 16 | 0.602 | 0.014 | 16 | 0.147 | 0.587 |
| Marans | 5 | 1 | ** | 5 | 1 | ** | 5 | 0.00 | 1.00 |
| Ohiki | 9 | 0.344 | 0.365 | 9 | 0.335 | 0.379 | 9 | 0.391 | 0.298 |
| Poland | 13 | 0.836 | < 0.001 | 13 | 0.972 | < 0.001 | 13 | -0.241 | 0.427 |
| Rosecomb bantam | 6 | -0.232 | 0.658 | 6 | 1 | ** | 6 | -0.926 | 0.008 |
| Yokohama | 6 | 0.985 | < 0.001 | 6 | 1 | < 0.001 | 6 | * | * |

* cannot be calculated because at least one of the variables is constant

** The correlation is significant at the 0.01 level (two-sided).
