# Supplementary material for: Differences among domestic chicken breeds in tonic immobility responses as a measure of fearfulness
Source: PeerJ. 2023 Apr 4;11:e14703. doi: 10.7717/peerj.14703 (PMC10081456; doi:10.7717/peerj.14703)
Supplement: Supplemental Information 3 — The analysis of the Tonic immobility (TI) response of mature hens of different breeds during repeated testing (part B) is given with the fixed effect breed and random effect age. p-values are marked with * for significances ( α-level was set at p ≤ 0.05 and indicated as *, p ≤ 0.01 is indicated as ** and p ≤ 0.001 as ***). Only significant pairwise comparisons (p ≤ 0.05) are highlighted green. [file peerj-11-14703-s003.docx]

**Supplemental table S3**

PART B:

**1^st^ Head movement x Breed**

Holm-Bonferroni post-hoc tests for pairwise comparisons of breeds for the variable **first head movement** (over all three repetitions) of mature hens of different breeds (part B). Given are the estimate, which indicates the difference between the pair (first minus second), standard error (SE) and the p-value. P-values are marked with * for significances (α-level was set at p ≤ 0.05 and indicated as *, p ≤ 0.01 is indicated as ** and p ≤ 0.001 as ***). Only significant pairwise comparisons (p ≤ 0.05) are highlighted green.

| **Breeds** | **Estimate** | **SE** | **p-value** |
| --- | --- | --- | --- |
| SI - BLC | 0.505 | 0.556 | 0.996 |
| SI - BS | 1.035 | 0.638 | 1 |
| SI - BR | 1.185 | 0.607 | 1 |
| SI - CB | 0.301 | 0.638 | 0.857 |
| SI - CO | 1.349 | 0.536 | 1 |
| SI - EFG | 0.822 | 0.607 | 1 |
| SI - GC | 0.315 | 0.547 | 0.723 |
| SI - JB | 1.814 | 0.510 | 0.998 |
| SI - LG | 8.718 | 0.607 | 0.038* |
| SI - LSL | 2.203 | 0.607 | 0.993 |
| SI - MA | 1.376 | 0.638 | 1 |
| SI - OH | 3.121 | 0.554 | 0.762 |
| SI - PO | 0.979 | 0.517 | 1 |
| SI - YO | 1.047 | 0.638 | 1 |
| BLC - BS | 2.048 | 0.601 | 0.997 |
| BLC - BR | 2.344 | 0.568 | 0.974 |
| BLC - CB | 0.596 | 0.601 | 1 |
| BLC - CO | 2.669 | 0.491 | 0.795 |
| BLC - EFG | 1.626 | 0.568 | 1 |
| BLC - GC | 0.622 | 0.503 | 1 |
| BLC - JB | 3.588 | 0.462 | 0.283 |
| BLC - LG | 17.248 | 0.568 | ≤ 0.001*** |
| BLC - LSL | 4.359 | 0.568 | 0.388 |
| BLC - MA | 2.722 | 0.601 | 0.938 |
| BLC - OH | 6.176 | 0.511 | 0.039* |
| BLC - PO | 1.938 | 0.470 | 0.985 |
| BLC - YO | 2.071 | 0.601 | 0.996 |
| BS - BR | 1.145 | 0.648 | 1 |
| BS - CB | 0.291 | 0.677 | 0.883 |
| BS - CO | 1.303 | 0.582 | 1 |
| BS - EFG | 0.794 | 0.648 | 1 |
| BS - GC | 0.304 | 0.592 | 0.787 |
| BS - JB | 1.752 | 0.558 | 1 |
| BS - LG | 8.423 | 0.648 | 0.085 |
| BS - LSL | 2.128 | 0.648 | 0.998 |
| BS - MA | 1.329 | 0.677 | 1 |
| BS - OH | 3.016 | 0.599 | 0.874 |
| BS - PO | 0.946 | 0.564 | 1 |
| BS - YO | 1.011 | 0.677 | 1 |
| BR - CB | 0.254 | 0.648 | 0.723 |
| BR - CO | 1.138 | 0.548 | 1 |
| BR - EFG | 0.693 | 0.618 | 1 |
| BR - GC | 0.266 | 0.559 | 0.540 |
| BR - JB | 1.531 | 0.522 | 1 |
| BR - LG | 7.357 | 0.618 | 0.099 |
| BR - LSL | 1.859 | 0.618 | 1 |
| BR - MA | 1.161 | 0.648 | 1 |
| BR - OH | 2.635 | 0.566 | 0.925 |
| BR - PO | 0.826 | 0.529 | 1 |
| BR - YO | 0.883 | 0.648 | 1 |
| CB - CO | 4.480 | 0.582 | 0.398 |
| CB - EFG | 2.729 | 0.648 | 0.965 |
| CB - GC | 1.045 | 0.592 | 1 |
| CB - JB | 6.022 | 0.558 | 0.101 |
| CB - LG | 28.951 | 0.648 | ≤ 0.001*** |
| CB - LSL | 7.316 | 0.648 | 0.146 |
| CB - MA | 4.569 | 0.677 | 0.633 |
| CB - OH | 10.367 | 0.599 | 0.013* |
| CB - PO | 3.252 | 0.564 | 0.738 |
| CB - YO | 3.476 | 0.677 | 0.875 |
| CO- EFG | 0.609 | 0.548 | 1 |
| CO- GC | 0.233 | 0.481 | 0.160 |
| CO- JB | 1.344 | 0.438 | 1 |
| CO- LG | 6.462 | 0.548 | 0.062 |
| CO- LSL | 1.633 | 0.548 | 1 |
| CO- MA | 1.020 | 0.582 | 1 |
| CO- OH | 2.314 | 0.489 | 0.924 |
| CO- PO | 0.726 | 0.446 | 1 |
| CO- YO | 0.776 | 0.582 | 1 |
| EFG - GC | 0.383 | 0.559 | 0.923 |
| EFG - JB | 2.207 | 0.522 | 0.971 |
| EFG - LG | 10.610 | 0.618 | 0.018* |
| EFG - LSL | 2.681 | 0.618 | 0.956 |
| EFG - MA | 1.675 | 0.648 | 1 |
| EFG - OH | 3.799 | 0.566 | 0.551 |
| EFG - PO | 1.192 | 0.529 | 1 |
| EFG - YO | 1.274 | 0.648 | 1 |
| GC - JB | 5.764 | 0.451 | 0.014* |
| GC - LG | 27.710 | 0.559 | ≤ 0.001*** |
| GC - LSL | 7.003 | 0.559 | 0.049* |
| GC - MA | 4.374 | 0.592 | 0.455 |
| GC - OH | 9.922 | 0.501 | 0.001*** |
| GC - PO | 3.112 | 0.459 | 0.467 |
| GC - YO | 3.327 | 0.592 | 0.776 |
| JB - LG | 4.807 | 0.522 | 0.170 |
| JB - LSL | 1.215 | 0.522 | 1 |
| JB - MA | 0.759 | 0.558 | 1 |
| JB - OH | 1.721 | 0.460 | 0.997 |
| JB - PO | 0.540 | 0.413 | 0.975 |
| JB - YO | 0.577 | 0.558 | 1 |
| LG - LSL | 0.253 | 0.618 | 0.646 |
| LG - MA | 0.158 | 0.648 | 0.240 |
| LG - OH | 0.358 | 0.566 | 0.887 |
| LG - PO | 0.112 | 0.529 | 0.006** |
| LG - YO | 0.120 | 0.648 | 0.089 |
| LSL - MA | 0.625 | 0.648 | 1 |
| LSL - OH | 1.417 | 0.566 | 1 |
| LSL - PO | 0.445 | 0.529 | 0.968 |
| LSL - YO | 0.475 | 0.648 | 0.998 |
| MA - OH | 2.269 | 0.599 | 0.988 |
| MA - PO | 0.712 | 0.564 | 1 |
| MA - YO | 0.761 | 0.677 | 1 |
| OH - PO | 0.314 | 0.467 | 0.464 |
| OH - YO | 0.335 | 0.599 | 0.882 |
| PO - YO | 1.069 | 0.564 | 1 |

**1^st^ Leg movement x Breed**

Holm-Bonferroni post-hoc tests for pairwise comparisons of breeds for the variable **first leg movement** (over all three repetitions) of mature hens of different breeds (part B). Given are the estimate, which indicates the difference between the pair (first minus second), standard error (SE) and the p-value. P-values are marked with * for significances (α-level was set at p ≤ 0.05 and indicated as *, p ≤ 0.01 is indicated as ** and p ≤ 0.001 as ***). Only significant pairwise comparisons (p ≤ 0.05) are highlighted green.

| **Breeds** | **Estimate** | **SE** | ***p*-value** |
| --- | --- | --- | --- |
| SI - BLC | 0.443 | 0.441 | 0.872 |
| SI - BS | 0.521 | 0.511 | 0.994 |
| SI - BR | 0.871 | 0.486 | 1 |
| SI - CB | 0.298 | 0.511 | 0.541 |
| SI - CO | 1.223 | 0.423 | 1 |
| SI - EFG | 0.571 | 0.486 | 0.998 |
| SI - GC | 0.280 | 0.437 | 0.210 |
| SI - JB | 1.470 | 0.405 | 1 |
| SI - LG | 2.327 | 0.486 | 0.916 |
| SI - LSL | 1.699 | 0.486 | 0.999 |
| SI - MA | 1.082 | 0.511 | 1 |
| SI - OH | 2.528 | 0.441 | 0.729 |
| SI - PO | 0.743 | 0.410 | 1 |
| SI - YO | 1.008 | 0.511 | 1 |
| BLC - BS | 1.177 | 0.484 | 1 |
| BLC - BR | 1.968 | 0.457 | 0.976 |
| BLC - CB | 0.672 | 0.484 | 1 |
| BLC - CO | 2.762 | 0.390 | 0.379 |
| BLC - EFG | 1.290 | 0.457 | 1 |
| BLC - GC | 0.633 | 0.405 | 0.998 |
| BLC - JB | 3.320 | 0.371 | 0.096 |
| BLC - LG | 5.254 | 0.457 | 0.032* |
| BLC - LSL | 3.835 | 0.457 | 0.197 |
| BLC - MA | 2.442 | 0.484 | 0.874 |
| BLC - OH | 5.706 | 0.409 | 0.004** |
| BLC - PO | 1.676 | 0.376 | 0.988 |
| BLC - YO | 2.277 | 0.484 | 0.928 |
| BS - BR | 1.672 | 0.525 | 1 |
| BS - CB | 0.571 | 0.549 | 0.999 |
| BS - CO | 2.348 | 0.468 | 0.883 |
| BS - EFG | 1.096 | 0.525 | 1 |
| BS - GC | 0.538 | 0.481 | 0.993 |
| BS - JB | 2.822 | 0.452 | 0.597 |
| BS - LG | 4.466 | 0.525 | 0.239 |
| BS - LSL | 3.260 | 0.525 | 0.629 |
| BS - MA | 2.075 | 0.549 | 0.991 |
| BS - OH | 4.850 | 0.484 | 0.090 |
| BS - PO | 1.425 | 0.457 | 1 |
| BS - YO | 1.935 | 0.549 | 0.997 |
| BR - CB | 0.342 | 0.525 | 0.767 |
| BR - CO | 1.404 | 0.440 | 1 |
| BR - EFG | 0.656 | 0.501 | 1 |
| BR - GC | 0.322 | 0.454 | 0.451 |
| BR - JB | 1.687 | 0.423 | 0.996 |
| BR - LG | 2.670 | 0.501 | 0.816 |
| BR - LSL | 1.949 | 0.501 | 0.991 |
| BR - MA | 1.241 | 0.525 | 1 |
| BR - OH | 2.900 | 0.457 | 0.572 |
| BR - PO | 0.852 | 0.428 | 1 |
| BR - YO | 1.157 | 0.525 | 1 |
| CB - CO | 4.110 | 0.468 | 0.164 |
| CB - EFG | 1.919 | 0.525 | 0.995 |
| CB - GC | 0.942 | 0.481 | 1 |
| CB - JB | 4.940 | 0.452 | 0.042* |
| CB - LG | 7.818 | 0.525 | 0.013* |
| CB - LSL | 5.706 | 0.525 | 0.079 |
| CB - MA | 3.633 | 0.549 | 0.556 |
| CB - OH | 8.491 | 0.484 | 0.002** |
| CB - PO | 2.495 | 0.457 | 0.792 |
| CB - YO | 3.388 | 0.549 | 0.648 |
| CO- EFG | 0.467 | 0.440 | 0.919 |
| CO- GC | 0.229 | 0.386 | 0.018* |
| CO- JB | 1.202 | 0.350 | 1 |
| CO- LG | 1.902 | 0.440 | 0.979 |
| CO- LSL | 1.388 | 0.440 | 1 |
| CO- MA | 0.884 | 0.468 | 1 |
| CO- OH | 2.066 | 0.390 | 0.866 |
| CO- PO | 0.607 | 0.355 | 0.985 |
| CO- YO | 0.824 | 0.468 | 1 |
| EFG - GC | 0.491 | 0.454 | 0.962 |
| EFG - JB | 2.574 | 0.423 | 0.641 |
| EFG - LG | 4.074 | 0.501 | 0.262 |
| EFG - LSL | 2.973 | 0.501 | 0.681 |
| EFG - MA | 1.893 | 0.525 | 0.996 |
| EFG - OH | 4.424 | 0.457 | 0.093 |
| EFG - PO | 1.300 | 0.428 | 1 |
| EFG - YO | 1.765 | 0.525 | 0.999 |
| GC - JB | 5.246 | 0.367 | 0.002** |
| GC - LG | 8.302 | 0.454 | ≤ 0.001*** |
| GC - LSL | 6.060 | 0.454 | 0.011* |
| GC - MA | 3.858 | 0.481 | 0.260 |
| GC - OH | 9.017 | 0.405 | ≤ 0.001*** |
| GC - PO | 2.649 | 0.372 | 0.371 |
| GC - YO | 3.597 | 0.481 | 0.343 |
| JB - LG | 1.583 | 0.423 | 0.999 |
| JB - LSL | 1.155 | 0.423 | 1 |
| JB - MA | 0.736 | 0.452 | 1 |
| JB - OH | 1.719 | 0.371 | 0.979 |
| JB - PO | 0.505 | 0.334 | 0.767 |
| JB - YO | 0.686 | 0.452 | 1 |
| LG - LSL | 0.730 | 0.501 | 1 |
| LG - MA | 0.465 | 0.525 | 0.979 |
| LG - OH | 1.086 | 0.457 | 1 |
| LG - PO | 0.319 | 0.428 | 0.340 |
| LG - YO | 0.433 | 0.525 | 0.957 |
| LSL - MA | 0.637 | 0.525 | 1 |
| LSL - OH | 1.488 | 0.457 | 1 |
| LSL - PO | 0.437 | 0.428 | 0.831 |
| LSL - YO | 0.594 | 0.525 | 1 |
| MA - OH | 2.337 | 0.484 | 0.910 |
| MA - PO | 0.687 | 0.457 | 1 |
| MA - YO | 0.932 | 0.549 | 1 |
| OH - PO | 0.294 | 0.376 | 0.092 |
| OH - YO | 0.399 | 0.484 | 0.848 |
| PO - YO | 1.358 | 0.457 | 1 |

**TI Duration x Breed**

Holm-Bonferroni post-hoc tests for pairwise comparisons of breeds for the variable **TI duration** (over all three repetitions) of mature hens of different breeds (part B). Given are the estimate, which indicates the difference between the pair (first minus second), standard error (SE) and the p-value. P-values are marked with * for significances (α-level was set at p ≤ 0.05 and indicated as *, p ≤ 0.01 is indicated as ** and p ≤ 0.001 as ***). Only significant pairwise comparisons (p ≤ 0.05) are highlighted green.

| **Breeds** | **Estimate** | **SE** | ***p*-value** |
| --- | --- | --- | --- |
| SI - BLC | 34.563 | 2.220 | 0.351 |
| SI - BS | 12.419 | 2.580 | 0.988 |
| SI - BR | 5.072 | 2.450 | 1 |
| SI - CB | 65.432 | 2.580 | 0.124 |
| SI - CO | 0.412 | 2.130 | 1 |
| SI - EFG | 29.117 | 2.450 | 0.662 |
| SI - GC | 71.809 | 2.170 | 0.013* |
| SI - JB | 1.560 | 2.040 | 1 |
| SI - LG | 0.075 | 2.450 | 1 |
| SI - LSL | 17.264 | 2.450 | 0.930 |
| SI - MA | 0.552 | 2.580 | 1 |
| SI - OH | 6.355 | 2.220 | 0.998 |
| SI - PO | 10.576 | 2.060 | 0.960 |
| SI - YO | 1.006 | 2.580 | 1 |
| BLC - BS | 5.546 | 2.460 | 1 |
| BLC - BR | 13.162 | 2.320 | 0.963 |
| BLC - CB | 4.884 | 2.460 | 1 |
| BLC - CO | 27.426 | 1.980 | 0.353 |
| BLC - EFG | 0.233 | 2.320 | 1 |
| BLC - GC | 6.729 | 2.020 | 0.994 |
| BLC - JB | 21.446 | 1.880 | 0.477 |
| BLC - LG | 31.427 | 2.320 | 0.509 |
| BLC - LSL | 100.681 | 2.320 | 0.003** |
| BLC - MA | 26.378 | 2.460 | 0.737 |
| BLC - OH | 70.560 | 2.080 | 0.008** |
| BLC - PO | 6.901 | 1.910 | 0.988 |
| BLC - YO | 23.775 | 2.460 | 0.802 |
| BS - BR | 1.621 | 2.670 | 1 |
| BS - CB | 20.839 | 2.780 | 0.946 |
| BS - CO | 8.306 | 2.370 | 0.996 |
| BS - EFG | 3.504 | 2.670 | 1 |
| BS - GC | 24.493 | 2.410 | 0.762 |
| BS - JB | 5.176 | 2.290 | 1 |
| BS - LG | 10.569 | 2.670 | 0.996 |
| BS - LSL | 58.967 | 2.670 | 0.224 |
| BS - MA | 7.734 | 2.780 | 1 |
| BS - OH | 36.542 | 2.460 | 0.477 |
| BS - PO | 0.074 | 2.320 | 1 |
| BS - YO | 6.355 | 2.780 | 1 |
| BR - CB | 34.071 | 2.670 | 0.671 |
| BR - CO | 2.589 | 2.230 | 1 |
| BR - EFG | 9.891 | 2.540 | 0.996 |
| BR - GC | 38.713 | 2.270 | 0.299 |
| BR - JB | 1.006 | 2.150 | 1 |
| BR - LG | 3.912 | 2.540 | 1 |
| BR - LSL | 41.050 | 2.540 | 0.436 |
| BR - MA | 2.274 | 2.670 | 1 |
| BR - OH | 22.772 | 2.320 | 0.759 |
| BR - PO | 1 | 2.170 | 1 |
| BR - YO | 1.560 | 2.670 | 1 |
| CB - CO | 55.458 | 2.370 | 0.124 |
| CB - EFG | 7.252 | 2.670 | 1 |
| CB - GC | 0.148 | 2.410 | 1 |
| CB - JB | 46.786 | 2.290 | 0.179 |
| CB - LG | 61.090 | 2.670 | 0.200 |
| CB - LSL | 149.916 | 2.670 | ≤ 0.001*** |
| CB - MA | 53.964 | 2.780 | 0.358 |
| CB - OH | 112.572 | 2.460 | 0.003** |
| CB - PO | 23.397 | 2.320 | 0.740 |
| CB - YO | 50.211 | 2.780 | 0.419 |
| CO- EFG | 22.601 | 2.230 | 0.714 |
| CO- GC | 61.325 | 1.920 | 0.008** |
| CO- JB | 0.367 | 1.770 | 1 |
| CO- LG | 0.136 | 2.230 | 1 |
| CO- LSL | 23.021 | 2.230 | 0.701 |
| CO- MA | 0.010 | 2.370 | 1 |
| CO- OH | 10.005 | 1.980 | 0.955 |
| CO- PO | 6.807 | 1.800 | 0.981 |
| CO- YO | 0.130 | 2.370 | 1 |
| EFG - GC | 9.474 | 2.270 | 0.989 |
| EFG - JB | 17.198 | 2.150 | 0.832 |
| EFG - LG | 26.245 | 2.540 | 0.785 |
| EFG - LSL | 91.222 | 2.540 | 0.021* |
| EFG - MA | 21.650 | 2.670 | 0.913 |
| EFG - OH | 62.679 | 2.320 | 0.060 |
| EFG - PO | 4.597 | 2.170 | 1 |
| EFG - YO | 19.298 | 2.670 | 0.943 |
| GC - JB | 52.201 | 1.820 | 0.011* |
| GC - LG | 67.240 | 2.270 | 0.034* |
| GC - LSL | 159.492 | 2.270 | ≤ 0.001*** |
| GC - MA | 59.753 | 2.410 | 0.104 |
| GC - OH | 120.868 | 2.020 | ≤ 0.001*** |
| GC - PO | 27.269 | 1.850 | 0.254 |
| GC - YO | 55.816 | 2.410 | 0.136 |
| JB - LG | 0.951 | 2.150 | 1 |
| JB - LSL | 29.203 | 2.150 | 0.439 |
| JB - MA | 0.255 | 2.290 | 1 |
| JB - OH | 14.205 | 1.880 | 0.791 |
| JB - PO | 4.012 | 1.700 | 0.997 |
| JB - YO | 0.061 | 2.290 | 1 |
| LG - LSL | 19.616 | 2.540 | 0.914 |
| LG - MA | 0.221 | 2.670 | 1 |
| LG - OH | 7.806 | 2.320 | 0.997 |
| LG - PO | 8.868 | 2.170 | 0.988 |
| LG - YO | 0.533 | 2.670 | 1 |
| LSL - MA | 24.000 | 2.670 | 0.877 |
| LSL - OH | 2.670 | 2.320 | 1 |
| LSL - PO | 54.864 | 2.170 | 0.061 |
| LSL - YO | 26.605 | 2.670 | 0.830 |
| MA - OH | 10.654 | 2.460 | 0.991 |
| MA - PO | 6.290 | 2.320 | 0.999 |
| MA - YO | 0.068 | 2.780 | 1 |
| OH - PO | 33.328 | 1.910 | 0.163 |
| OH - YO | 12.419 | 2.460 | 0.982 |
| PO - YO | 5.058 | 2.320 | 1 |

**Number of attempts x Breed**

Holm-Bonferroni post-hoc tests for pairwise comparisons of breeds for the variable **number of attempts to induce TI** (over all three repetitions) of mature hens of different breeds (part B). Given are the estimate, which indicates the difference between the pair (first minus second), standard error (SE) and the p-value. P-values are marked with * for significances (α-level was set at p ≤ 0.05 and indicated as *, p ≤ 0.01 is indicated as ** and p ≤ 0.001 as ***). Only significant pairwise comparisons (p ≤ 0.05) are highlighted green.

| **Breeds** | **Estimate** | **SE** | **p-value** |
| --- | --- | --- | --- |
| SI - BLC | 0.962 | 0.354 | 1 |
| SI - BS | 1.140 | 0.420 | 1 |
| SI - BR | 1.008 | 0.392 | 1 |
| SI - CB | 1.367 | 0.433 | 1 |
| SI - CO | 1.105 | 0.344 | 1 |
| SI - EFG | 1.072 | 0.395 | 1 |
| SI - GC | 1.368 | 0.360 | 1 |
| SI - JB | 1.072 | 0.328 | 1 |
| SI - LG | 0.769 | 0.380 | 1 |
| SI - LSL | 0.949 | 0.389 | 1 |
| SI - MA | 1.084 | 0.417 | 1 |
| SI - OH | 0.391 | 0.332 | 0.232 |
| SI - PO | 0.634 | 0.321 | 0.986 |
| SI - YO | 1.205 | 0.424 | 1 |
| BLC - BS | 1.185 | 0.400 | 1 |
| BLC - BR | 1.048 | 0.370 | 1 |
| BLC - CB | 1.422 | 0.414 | 1 |
| BLC - CO | 1.149 | 0.319 | 1 |
| BLC - EFG | 1.115 | 0.374 | 1 |
| BLC - GC | 1.423 | 0.336 | 0.999 |
| BLC - JB | 1.115 | 0.302 | 1 |
| BLC - LG | 0.800 | 0.357 | 1 |
| BLC - LSL | 0.987 | 0.367 | 1 |
| BLC - MA | 1.127 | 0.397 | 1 |
| BLC - OH | 0.406 | 0.306 | 0.177 |
| BLC - PO | 0.659 | 0.294 | 0.986 |
| BLC - YO | 1.253 | 0.404 | 1 |
| BS - BR | 0.884 | 0.435 | 1 |
| BS - CB | 1.200 | 0.472 | 1 |
| BS - CO | 0.970 | 0.392 | 1 |
| BS - EFG | 0.941 | 0.438 | 1 |
| BS - GC | 1.200 | 0.406 | 1 |
| BS - JB | 0.941 | 0.378 | 1 |
| BS - LG | 0.675 | 0.423 | 1 |
| BS - LSL | 0.833 | 0.432 | 1 |
| BS - MA | 0.951 | 0.457 | 1 |
| BS - OH | 0.343 | 0.382 | 0.245 |
| BS - PO | 0.556 | 0.372 | 0.964 |
| BS - YO | 1.057 | 0.464 | 1 |
| BR - CB | 1.356 | 0.447 | 1 |
| BR - CO | 1.096 | 0.361 | 1 |
| BR - EFG | 1.063 | 0.411 | 1 |
| BR - GC | 1.357 | 0.376 | 1 |
| BR - JB | 1.064 | 0.346 | 1 |
| BR - LG | 0.763 | 0.395 | 1 |
| BR - LSL | 0.942 | 0.404 | 1 |
| BR - MA | 1.076 | 0.432 | 1 |
| BR - OH | 0.388 | 0.350 | 0.301 |
| BR - PO | 0.629 | 0.340 | 0.990 |
| BR - YO | 1.195 | 0.438 | 1 |
| CB - CO | 0.808 | 0.406 | 1 |
| CB - EFG | 0.784 | 0.450 | 1 |
| CB - GC | 1.001 | 0.419 | 1 |
| CB - JB | 0.784 | 0.392 | 1 |
| CB - LG | 0.562 | 0.436 | 0.993 |
| CB - LSL | 0.694 | 0.445 | 1 |
| CB - MA | 0.793 | 0.469 | 1 |
| CB - OH | 0.286 | 0.396 | 0.099 |
| CB - PO | 0.463 | 0.387 | 0.806 |
| CB - YO | 0.881 | 0.476 | 1 |
| CO- EFG | 0.970 | 0.365 | 1 |
| CO- GC | 1.238 | 0.326 | 1 |
| CO- JB | 0.970 | 0.290 | 1 |
| CO- LG | 0.696 | 0.348 | 0.999 |
| CO- LSL | 0.859 | 0.358 | 1 |
| CO- MA | 0.981 | 0.388 | 1 |
| CO- OH | 0.354 | 0.295 | 0.033* |
| CO- PO | 0.573 | 0.283 | 0.818 |
| CO- YO | 1.090 | 0.396 | 1 |
| EFG - GC | 1.276 | 0.380 | 1 |
| EFG - JB | 1 | 0.350 | 1 |
| EFG - LG | 0.717 | 0.399 | 1 |
| EFG - LSL | 0.885 | 0.408 | 1 |
| EFG - MA | 1.011 | 0.435 | 1 |
| EFG - OH | 0.365 | 0.354 | 0.220 |
| EFG - PO | 0.591 | 0.344 | 0.972 |
| EFG - YO | 1.124 | 0.441 | 1 |
| GC - JB | 0.784 | 0.309 | 1 |
| GC - LG | 0.562 | 0.363 | 0.962 |
| GC - LSL | 0.694 | 0.373 | 1 |
| GC - MA | 0.793 | 0.402 | 1 |
| GC - OH | 0.286 | 0.314 | 0.006** |
| GC - PO | 0.463 | 0.302 | 0.405 |
| GC - YO | 0.881 | 0.410 | 1 |
| JB - LG | 0.717 | 0.332 | 1 |
| JB - LSL | 0.885 | 0.342 | 1 |
| JB - MA | 1.011 | 0.374 | 1 |
| JB - OH | 0.364 | 0.276 | 0.021* |
| JB - PO | 0.591 | 0.263 | 0.800 |
| JB - YO | 1.124 | 0.382 | 1 |
| LG - LSL | 1.235 | 0.392 | 1 |
| LG - MA | 1.410 | 0.420 | 1 |
| LG - OH | 0.508 | 0.336 | 0.791 |
| LG - PO | 0.824 | 0.325 | 1 |
| LG - YO | 1.567 | 0.427 | 0.999 |
| LSL - MA | 1.142 | 0.429 | 1 |
| LSL - OH | 0.412 | 0.347 | 0.398 |
| LSL - PO | 0.667 | 0.336 | 0.997 |
| LSL - YO | 1.269 | 0.436 | 1 |
| MA - OH | 0.360 | 0.378 | 0.306 |
| MA - PO | 0.584 | 0.369 | 0.982 |
| MA - YO | 1.111 | 0.461 | 1 |
| OH - PO | 1.621 | 0.267 | 0.896 |
| OH - YO | 3.083 | 0.386 | 0.188 |
| PO - YO | 1.902 | 0.377 | 0.932 |
