# Supplementary material for: Differences among domestic chicken breeds in tonic immobility responses as a measure of fearfulness
Source: PeerJ. 2023 Apr 4;11:e14703. doi: 10.7717/peerj.14703 (PMC10081456; doi:10.7717/peerj.14703)
Supplement: Supplemental Information 5 — A power analysis has been conducted on the complete data set to indicate sufficient sample sizes or underrepresented chicken breeds. [file peerj-11-14703-s005.docx]

**Table S5: Power-analysis for** **subset A:** Post hoc calculation of the required sample size (for the correlations between the variables within each breed) that would be required to detect a significant effect.
Given are the actual sample size (actual n) of each breed as well as the input parameters of the power analysis: one- or two-sided (tails), the correlation coefficient of the actual correlation (r H1; see table S5), the probability of α-error (α-error), the power of the test (power), the correlation coefficient of the null hypothesis (r H0) and the calculation of the sample size that would be required to show a significant effect (required n). The instances where required sample sizes were met are marked in green.

| **Subset A** |  | **G*Power-analysis for First Head Movement - Turn** | | | | | |
| --- | --- | --- | --- | --- | --- | --- | --- |
| **Breed** | actual n | tails | r H1 | α-error | power | r H0 | required n |
| Bantam Silkie | 6 | two-sided | 0.943 | 0.05 | 0.8 | 0 | 5 |
| Bergische Long Crower | 9 | two-sided | 0.945 | 0.05 | 0.8 | 0 | 5 |
| Bergische Schlotterkämm | 17 | two-sided | 0.663 | 0.05 | 0.8 | 0 | 15 |
| Breda | 8 | two-sided | 0.994 | 0.05 | 0.8 | 0 | 4 |
| Cobb 500 | 5 | two-sided | 1 | 0.05 | 0.8 | 0 | 3 |
| Cochin | 12 | two-sided | 0.818 | 0.05 | 0.8 | 0 | 9 |
| East Frisian Gull | 6 | two-sided | 0.274 | 0.05 | 0.8 | 0 | 102 |
| German Creeper | 10 | two-sided | 0.982 | 0.05 | 0.8 | 0 | 4 |
| German Empire Breed | 10 | two-sided | 0.867 | 0.05 | 0.8 | 0 | 7 |
| Japanese bantam | 16 | two-sided | 0.846 | 0.05 | 0.8 | 0 | 8 |
| Leghorn | 6 | two-sided | 0.600 | 0.05 | 0.8 | 0 | 19 |
| Lohmann Brown | 10 | two-sided | 0.395 | 0.05 | 0.8 | 0 | 47 |
| Lohmann Dual | 7 | two-sided | 0.296 | 0.05 | 0.8 | 0 | 26 |
| LSL | 16 | two-sided | 0.439 | 0.05 | 0.8 | 0 | 38 |
| Marans | 5 | two-sided | 1 | 0.05 | 0.8 | 0 | 3 |
| Ohiki | 9 | two-sided | 0.344 | 0.05 | 0.8 | 0 | 64 |
| Poland | 13 | two-sided | 0.836 | 0.05 | 0.8 | 0 | 8 |
| Rosecomb bantam | 6 | two-sided | -0.232 | 0.05 | 0.8 | 0 | 143 |
| Yokohama | 6 | two-sided | 0.985 | 0.05 | 0.8 | 0 | 4 |
| **Subset A** |  | **G*Power-analysis for First Leg Movement - Turn** | | | | | |
| **Breed** | actual n | tails | r H1 | α-error | power | r H0 | required n |
| Bantam Silkie | 7 | two-sided | 0.964 | 0.05 | 0.8 | 0 | 5 |
| Bergische Long Crower | 9 | two-sided | 0.945 | 0.05 | 0.8 | 0 | 5 |
| Bergische Schlotterkämm | 17 | two-sided | 0.526 | 0.05 | 0.8 | 0 | 26 |
| Breda | 8 | two-sided | 1 | 0.05 | 0.8 | 0 | 3 |
| Cobb 500 | 5 | two-sided | 1 | 0.05 | 0.8 | 0 | 3 |
| Cochin | 12 | two-sided | 0.832 | 0.05 | 0.8 | 0 | 8 |
| East Frisian Gull | 6 | two-sided | 0.920 | 0.05 | 0.8 | 0 | 6 |
| German Creeper | 10 | two-sided | 1 | 0.05 | 0.8 | 0 | 3 |
| German Empire Breed | 10 | two-sided | 0.955 | 0.05 | 0.8 | 0 | 5 |
| Japanese bantam | 16 | two-sided | 0.893 | 0.05 | 0.8 | 0 | 7 |
| Leghorn | 6 | two-sided | 0.429 | 0.05 | 0.8 | 0 | 40 |
| Lohmann Brown | 10 | two-sided | 0.665 | 0.05 | 0.8 | 0 | 15 |
| Lohmann Dual | 7 | two-sided | 0.786 | 0.05 | 0.8 | 0 | 10 |
| LSL | 16 | two-sided | 0.602 | 0.05 | 0.8 | 0 | 19 |
| Marans | 5 | two-sided | 1 | 0.05 | 0.8 | 0 | 3 |
| Ohiki | 9 | two-sided | 0.335 | 0.05 | 0.8 | 0 | 67 |
| Poland | 13 | two-sided | 0.972 | 0.05 | 0.8 | 0 | 5 |
| Rosecomb bantam | 6 | two-sided | 1 | 0.05 | 0.8 | 0 | 3 |
| Yokohama | 6 | two-sided | 1 | 0.05 | 0.8 | 0 | 3 |
| **Subset A** |  | **G*Power-analysis for Number of inductions - Turn** | | | | | |
| **Breed** | actual n | tails | r H1 | α-error | power | r H0 | required n |
| Bantam Silkie | 7 | two-sided | 0.359 | 0.05 | 0.8 | 0 | 58 |
| Bergische Long Crower | 9 | two-sided | -0.429 | 0.05 | 0.8 | 0 | 40 |
| Bergische Schlotterkämm | 17 | two-sided | 0.358 | 0.05 | 0.8 | 0 | 58 |
| Breda | 8 | two-sided | -0.415 | 0.05 | 0.8 | 0 | 43 |
| Cobb 500 | 5 | two-sided | * | 0.05 | 0.8 | 0 | * |
| Cochin | 12 | two-sided | 0.218 | 0.05 | 0.8 | 0 | 162 |
| East Frisian Gull | 6 | two-sided | -0.845 | 0.05 | 0.8 | 0 | 8 |
| German Creeper | 10 | two-sided | * | 0.05 | 0.8 | 0 | * |
| German Empire Breed | 10 | two-sided | 0.725 | 0.05 | 0.8 | 0 | 12 |
| Japanese bantam | 16 | two-sided | -0.224 | 0.05 | 0.8 | 0 | 154 |
| Leghorn | 6 | two-sided | -0.169 | 0.05 | 0.8 | 0 | 272 |
| Lohmann Brown | 10 | two-sided | -0.174 | 0.05 | 0.8 | 0 | 256 |
| Lohmann Dual | 7 | two-sided | 0.00 | 0.05 | 0.8 | 0 | * |
| LSL | 16 | two-sided | 0.147 | 0.05 | 0.8 | 0 | 360 |
| Marans | 5 | two-sided | 0.00 | 0.05 | 0.8 | 0 | * |
| Ohiki | 9 | two-sided | 0.391 | 0.05 | 0.8 | 0 | 49 |
| Poland | 13 | two-sided | -0.241 | 0.05 | 0.8 | 0 | 132 |
| Rosecomb bantam | 6 | two-sided | -0.926 | 0.05 | 0.8 | 0 | 6 |
| Yokohama | 6 | two-sided | * | 0.05 | 0.8 | 0 | * |

* cannot be calculated because at least one of the variables is constant

**Power-analysis for** **subset B:** Post hoc calculation of the required sample size (for the correlations between the variables within each breed and within each repetition) that would be necessary to show a significant effect.
Given are the actual sample size (actual n) of each breed within repetition as well as the input parameters of the power analysis: one- or two-sided (tails), the correlation coefficient of the actual correlation (r H1; see table S5), the probability of α-error (α-error), the power of the test (power), the correlation coefficient of the null hypothesis (r H0) and the calculation of the sample size that would be required to show a significant effect (required n). The instances where required sample sizes were met are marked in green.

| **Subset B – 1^st^ repetition** |  | **G*Power-analysis for First Head Movement - Turn** | | | | | |
| --- | --- | --- | --- | --- | --- | --- | --- |
| **Breed** | actual n | tails | r H1 | α-error | power | r H0 | required n |
| Bantam Silkie | 7 | two-sided | 0.943 | 0.05 | 0.8 | 0 | 5 |
| Bergische Long Crower | 9 | two-sided | 0.945 | 0.05 | 0.8 | 0 | 5 |
| Bergische Schlotterkämm | 5 | two-sided | 1 | 0.05 | 0.8 | 0 | 3 |
| Breda | 6 | two-sided | 1 | 0.05 | 0.8 | 0 | 3 |
| Cobb 500 | 5 | two-sided | 1 | 0.05 | 0.8 | 0 | 3 |
| Cochin | 11 | two-sided | 0.764 | 0.05 | 0.8 | 0 | 10 |
| East Frisian Gull | 6 | two-sided | 0.086 | 0.05 | 0.8 | 0 | 1058 |
| German Creeper | 10 | two-sided | 0.982 | 0.05 | 0.8 | 0 | 4 |
| Japanese bantam | 14 | two-sided | 0.845 | 0.05 | 0.8 | 0 | 8 |
| Leghorn | 6 | two-sided | 0.600 | 0.05 | 0.8 | 0 | 19 |
| LSL | 6 | two-sided | 0.429 | 0.05 | 0.8 | 0 | 40 |
| Marans | 5 | two-sided | 1 | 0.05 | 0.8 | 0 | 3 |
| Ohiki | 9 | two-sided | 0.400 | 0.05 | 0.8 | 0 | 46 |
| Poland | 13 | two-sided | 0.836 | 0.05 | 0.8 | 0 | 8 |
| Yokohama | 5 | two-sided | 1 | 0.05 | 0.8 | 0 | 3 |
| **Subset B – 1^st^ repetition** |  | **G*Power-analysis for First Leg Movement - Turn** | | | | | |
| **Breed** | actual n | tails | r H1 | α-error | power | r H0 | required n |
| Bantam Silkie | 7 | two-sided | 0.964 | 0.05 | 0.8 | 0 | 5 |
| Bergische Long Crower | 9 | two-sided | 0.945 | 0.05 | 0.8 | 0 | 5 |
| Bergische Schlotterkämm | 5 | two-sided | 1 | 0.05 | 0.8 | 0 | 3 |
| Breda | 6 | two-sided | 1 | 0.05 | 0.8 | 0 | 3 |
| Cobb 500 | 5 | two-sided | 1 | 0.05 | 0.8 | 0 | 3 |
| Cochin | 11 | two-sided | 0.782 | 0.05 | 0.8 | 0 | 10 |
| East Frisian Gull | 6 | two-sided | 0.943 | 0.05 | 0.8 | 0 | 5 |
| German Creeper | 10 | two-sided | 1 | 0.05 | 0.8 | 0 | 3 |
| Japanese bantam | 14 | two-sided | 0.907 | 0.05 | 0.8 | 0 | 6 |
| Leghorn | 6 | two-sided | 0.429 | 0.05 | 0.8 | 0 | 40 |
| LSL | 6 | two-sided | 0.943 | 0.05 | 0.8 | 0 | 5 |
| Marans | 5 | two-sided | 1 | 0.05 | 0.8 | 0 | 3 |
| Ohiki | 9 | two-sided | 0.417 | 0.05 | 0.8 | 0 | 42 |
| Poland | 13 | two-sided | 0.972 | 0.05 | 0.8 | 0 | 5 |
| Yokohama | 5 | two-sided | 1 | 0.05 | 0.8 | 0 | 3 |
| **Subset B – 1^st^ repetition** |  | **G*Power-analysis for Number of inductions - Turn** | | | | | |
| **Breed** | actual n | tails | r H1 | α-error | power | r H0 | required n |
| Bantam Silkie | 7 | two-sided | 0.359 | 0.05 | 0.8 | 0 | 58 |
| Bergische Long Crower | 9 | two-sided | -0.429 | 0.05 | 0.8 | 0 | 40 |
| Bergische Schlotterkämm | 5 | two-sided | * | 0.05 | 0.8 | 0 | * |
| Breda | 6 | two-sided | -0.664 | 0.05 | 0.8 | 0 | 15 |
| Cobb 500 | 5 | two-sided | * | 0.05 | 0.8 | 0 | * |
| Cochin | 11 | two-sided | 0.300 | 0.05 | 0.8 | 0 | 84 |
| East Frisian Gull | 6 | two-sided | -0.845 | 0.05 | 0.8 | 0 | 8 |
| German Creeper | 10 | two-sided | * | 0.05 | 0.8 | 0 | * |
| Japanese bantam | 14 | two-sided | 0.001 | 0.05 | 0.8 | 0 | 7848859 |
| Leghorn | 6 | two-sided | -0.169 | 0.05 | 0.8 | 0 | 272 |
| LSL | 6 | two-sided | 0.338 | 0.05 | 0.8 | 0 | 66 |
| Marans | 5 | two-sided | 0.001 | 0.05 | 0.8 | 0 | 7848859 |
| Ohiki | 9 | two-sided | 0.374 | 0.05 | 0.8 | 0 | 53 |
| Poland | 13 | two-sided | -0.241 | 0.05 | 0.8 | 0 | 132 |
| Yokohama | 5 | two-sided | * | 0.05 | 0.8 | 0 | * |
| **Subset B – 2^nd^ repetition** |  | **G*Power-analysis for First Head Movement - Turn** | | | | | |
| **Breed** | actual n | tails | r H1 | α-error | power | r H0 | required n |
| Bantam Silkie | 7 | two-sided | 0.900 | 0.05 | 0.8 | 0 | 6 |
| Bergische Long Crower | 9 | two-sided | 0.957 | 0.05 | 0.8 | 0 | 5 |
| Bergische Schlotterkämm | 5 | two-sided | 0.600 | 0.05 | 0.8 | 0 | 19 |
| Breda | 6 | two-sided | 1 | 0.05 | 0.8 | 0 | 3 |
| Cobb 500 | 5 | two-sided | 1 | 0.05 | 0.8 | 0 | 3 |
| Cochin | 11 | two-sided | 0.798 | 0.05 | 0.8 | 0 | 9 |
| East Frisian Gull | 6 | two-sided | 0.086 | 0.05 | 0.8 | 0 | 1058 |
| German Creeper | 10 | two-sided | 1 | 0.05 | 0.8 | 0 | 3 |
| Japanese bantam | 14 | two-sided | 0.837 | 0.05 | 0.8 | 0 | 8 |
| Leghorn | 6 | two-sided | 0.657 | 0.05 | 0.8 | 0 | 15 |
| LSL | 6 | two-sided | 0.943 | 0.05 | 0.8 | 0 | 5 |
| Marans | 5 | two-sided | 0.895 | 0.05 | 0.8 | 0 | 6 |
| Ohiki | 9 | two-sided | 0.733 | 0.05 | 0.8 | 0 | 12 |
| Poland | 13 | two-sided | 0.836 | 0.05 | 0.8 | 0 | 8 |
| Yokohama | 5 | two-sided | 0.900 | 0.05 | 0.8 | 0 | 6 |
| **Subset B – 2^nd^ repetition** |  | **G*Power-analysis for First Leg Movement - Turn** | | | | | |
| **Breed** | actual n | tails | r H1 | α-error | power | r H0 | required n |
| Bantam Silkie | 7 | two-sided | 0.964 | 0.05 | 0.8 | 0 | 5 |
| Bergische Long Crower | 9 | two-sided | 1 | 0.05 | 0.8 | 0 | 3 |
| Bergische Schlotterkämm | 5 | two-sided | 1 | 0.05 | 0.8 | 0 | 3 |
| Breda | 6 | two-sided | 1 | 0.05 | 0.8 | 0 | 3 |
| Cobb 500 | 5 | two-sided | 1 | 0.05 | 0.8 | 0 | 3 |
| Cochin | 11 | two-sided | 0.808 | 0.05 | 0.8 | 0 | 9 |
| East Frisian Gull | 6 | two-sided | 0.200 | 0.05 | 0.8 | 0 | 193 |
| German Creeper | 10 | two-sided | 1 | 0.05 | 0.8 | 0 | 3 |
| Japanese bantam | 14 | two-sided | 0.262 | 0.05 | 0.8 | 0 | 112 |
| Leghorn | 6 | two-sided | 0.257 | 0.05 | 0.8 | 0 | 116 |
| LSL | 6 | two-sided | 1 | 0.05 | 0.8 | 0 | 3 |
| Marans | 5 | two-sided | 1 | 0.05 | 0.8 | 0 | 3 |
| Ohiki | 9 | two-sided | 0.833 | 0.05 | 0.8 | 0 | 8 |
| Poland | 13 | two-sided | 0.983 | 0.05 | 0.8 | 0 | 4 |
| Yokohama | 5 | two-sided | 0.900 | 0.05 | 0.8 | 0 | 6 |
| **Subset B – 2^nd^ repetition** |  | **G*Power-analysis for Number of inductions - Turn** | | | | | |
| **Breed** | actual n | tails | r H1 | α-error | power | r H0 | required n |
| Bantam Silkie | 7 | two-sided | -0.668 | 0.05 | 0.8 | 0 | 15 |
| Bergische Long Crower | 9 | two-sided | -0.620 | 0.05 | 0.8 | 0 | 18 |
| Bergische Schlotterkämm | 5 | two-sided | * | 0.05 | 0.8 | 0 | * |
| Breda | 6 | two-sided | -0.393 | 0.05 | 0.8 | 0 | 48 |
| Cobb 500 | 5 | two-sided | * | 0.05 | 0.8 | 0 | * |
| Cochin | 11 | two-sided | 0.284 | 0.05 | 0.8 | 0 | 95 |
| East Frisian Gull | 6 | two-sided | * | 0.05 | 0.8 | 0 | * |
| German Creeper | 10 | two-sided | * | 0.05 | 0.8 | 0 | * |
| Japanese bantam | 14 | two-sided | -0.284 | 0.05 | 0.8 | 0 | 95 |
| Leghorn | 6 | two-sided | 0.123 | 0.05 | 0.8 | 0 | 516 |
| LSL | 6 | two-sided | 0.507 | 0.05 | 0.8 | 0 | 28 |
| Marans | 5 | two-sided | 0.001 | 0.05 | 0.8 | 0 | 7848859 |
| Ohiki | 9 | two-sided | -0.256 | 0.05 | 0.8 | 0 | 117 |
| Poland | 13 | two-sided | -0.093 | 0.05 | 0.8 | 0 | 905 |
| Yokohama | 5 | two-sided | 0.001 | 0.05 | 0.8 | 0 | 7848859 |
| **Subset B – 3^rd^ repetition** |  | **G*Power-analysis for First Head Movement - Turn** | | | | | |
| **Breed** | actual n | tails | r H1 | α-error | power | r H0 | required n |
| Bantam Silkie | 7 | two-sided | 0.964 | 0.05 | 0.8 | 0 | 5 |
| Bergische Long Crower | 9 | two-sided | 1 | 0.05 | 0.8 | 0 | 3 |
| Bergische Schlotterkämm | 5 | two-sided | 0.579 | 0.05 | 0.8 | 0 | 21 |
| Breda | 6 | two-sided | 0.941 | 0.05 | 0.8 | 0 | 5 |
| Cobb 500 | 5 | two-sided | * | 0.05 | 0.8 | 0 | * |
| Cochin | 11 | two-sided | 0.879 | 0.05 | 0.8 | 0 | 7 |
| East Frisian Gull | 6 | two-sided | 0.371 | 0.05 | 0.8 | 0 | 54 |
| German Creeper | 10 | two-sided | 1 | 0.05 | 0.8 | 0 | 3 |
| Japanese bantam | 14 | two-sided | 0.793 | 0.05 | 0.8 | 0 | 9 |
| Leghorn | 6 | two-sided | 0.143 | 0.05 | 0.8 | 0 | 381 |
| LSL | 6 | two-sided | 0.771 | 0.05 | 0.8 | 0 | 10 |
| Marans | 5 | two-sided | 1 | 0.05 | 0.8 | 0 | 3 |
| Ohiki | 9 | two-sided | 0.833 | 0.05 | 0.8 | 0 | 8 |
| Poland | 13 | two-sided | 0.780 | 0.05 | 0.8 | 0 | 10 |
| Yokohama | 5 | two-sided | 1 | 0.05 | 0.8 | 0 | 3 |
| **Subset B – 3^rd^ repetition** |  | **G*Power-analysis for First Leg Movement - Turn** | | | | | |
| **Breed** | actual n | tails | r H1 | α-error | power | r H0 | required n |
| Bantam Silkie | 7 | two-sided | 0.771 | 0.05 | 0.8 | 0 | 10 |
| Bergische Long Crower | 9 | two-sided | 1 | 0.05 | 0.8 | 0 | 3 |
| Bergische Schlotterkämm | 5 | two-sided | 1 | 0.05 | 0.8 | 0 | 3 |
| Breda | 6 | two-sided | 0.941 | 0.05 | 0.8 | 0 | 5 |
| Cobb 500 | 5 | two-sided | * | 0.05 | 0.8 | 0 | * |
| Cochin | 11 | two-sided | 0.900 | 0.05 | 0.8 | 0 | 6 |
| East Frisian Gull | 6 | two-sided | 0.943 | 0.05 | 0.8 | 0 | 5 |
| German Creeper | 10 | two-sided | 0.976 | 0.05 | 0.8 | 0 | 4 |
| Japanese bantam | 14 | two-sided | 0.945 | 0.05 | 0.8 | 0 | 5 |
| Leghorn | 6 | two-sided | -0.029 | 0.05 | 0.8 | 0 | 9330 |
| LSL | 6 | two-sided | 1 | 0.05 | 0.8 | 0 | 3 |
| Marans | 5 | two-sided | 1 | 0.05 | 0.8 | 0 | 3 |
| Ohiki | 9 | two-sided | 0.917 | 0.05 | 0.8 | 0 | 6 |
| Poland | 13 | two-sided | 0.761 | 0.05 | 0.8 | 0 | 11 |
| Yokohama | 5 | two-sided | 1 | 0.05 | 0.8 | 0 | 3 |
| **Subset B – 3^rd^ repetition** |  | **G*Power-analysis for Number of inductions - Turn** | | | | | |
| **Breed** | actual n | tails | r H1 | α-error | power | r H0 | required n |
| Bantam Silkie | 7 | two-sided | * | 0.05 | 0.8 | 0 | * |
| Bergische Long Crower | 9 | two-sided | -0.050 | 0.05 | 0.8 | 0 | 3137 |
| Bergische Schlotterkämm | 5 | two-sided | 0.001 | 0.05 | 0.8 | 0 | 7848859 |
| Breda | 6 | two-sided | -0.399 | 0.05 | 0.8 | 0 | 46 |
| Cobb 500 | 5 | two-sided | * | 0.05 | 0.8 | 0 | * |
| Cochin | 11 | two-sided | -0.224 | 0.05 | 0.8 | 0 | 154 |
| East Frisian Gull | 6 | two-sided | -0.655 | 0.05 | 0.8 | 0 | 15 |
| German Creeper | 10 | two-sided | * | 0.05 | 0.8 | 0 | * |
| Japanese bantam | 14 | two-sided | 0.106 | 0.05 | 0.8 | 0 | 696 |
| Leghorn | 6 | two-sided | -0.845 | 0.05 | 0.8 | 0 | 8 |
| LSL | 6 | two-sided | * | 0.05 | 0.8 | 0 | * |
| Marans | 5 | two-sided | -0.707 | 0.05 | 0.8 | 0 | 13 |
| Ohiki | 9 | two-sided | -0.154 | 0.05 | 0.8 | 0 | 328 |
| Poland | 13 | two-sided | -0.127 | 0.05 | 0.8 | 0 | 484 |
| Yokohama | 5 | two-sided | -0.707 | 0.05 | 0.8 | 0 | 13 |
